# Genetically determined body mass index is associated with diffuse large B‐cell lymphoma in polygenic and Mendelian randomization analyses

**DOI:** 10.1002/ijc.70039

**Published:** 2025-09-05

**Authors:** Amy Moore, Eleanor Kane, Lauren R. Teras, Mitchell J. Machiela, Joshua Arias, Orestis A. Panagiotou, Alain Monnereau, Nicole Wong Doo, Zhaoming Wang, Susan L. Slager, Roel C. H. Vermeulen, Claire M. Vajdic, Karin E. Smedby, John J. Spinelli, Joseph Vijai, Graham G. Giles, Brian K. Link, Alan A. Arslan, Alexandra Nieters, Paige M. Bracci, Nicola J. Camp, Gilles Salles, Wendy Cozen, Henrik Hjalgrim, Immaculata De Vivo, Hans‐Olov Adami, Demetrius Albanes, Nikolaus Becker, Yolanda Benavente, Simonetta Bisanzi, Paolo Boffetta, Paul Brennan, Angela R. Brooks‐Wilson, Federico Canzian, Jacqueline Clavel, Lucia Conde, David G. Cox, Karen Curtin, Lenka Foretova, Hervé Ghesquières, Bengt Glimelius, Thomas M. Habermann, Jonathan N. Hofmann, Qing Lan, Mark Liebow, Anne Lincoln, Marc Maynadie, James McKay, Mads Melbye, Lucia Miligi, Roger L. Milne, Thierry J. Molina, Lindsay M. Morton, Kari E. North, Kenneth Offit, Marina Padoan, Sara Piro, Alpa V. Patel, Mark P. Purdue, Vignesh Ravichandran, Elio Riboli, Richard K. Severson, Melissa C. Southey, Anthony Staines, Lesley F. Tinker, Ruth C. Travis, Sophia S. Wang, Elisabete Weiderpass, Stephanie Weinstein, Tongzhang Zheng, Stephen J. Chanock, Nathaniel Rothman, Brenda M. Birmann, James R. Cerhan, Sonja I. Berndt

**Affiliations:** ^1^ Division of Cancer Epidemiology and Genetics National Cancer Institute Bethesda Maryland USA; ^2^ Department of Health Sciences University of York York UK; ^3^ Department of Population Science American Cancer Society Atlanta Georgia USA; ^4^ Department of Health Services, Policy, and Practice Brown University School of Public Health Providence Rhode Island USA; ^5^ Center for Gerontology and Healthcare Research Brown University School of Public Health Providence Rhode Island USA; ^6^ Unit of Mixed Research (UMR1153) Centre for Research in Epidemiology and Statistics (CRESS), INSERM, Université Paris‐Cité Paris France; ^7^ Registre des hémopathies malignes de la Gironde Institut Bergonié Bordeaux Cedex France; ^8^ Concord Clinical School University of Sydney Concord New South Wales Australia; ^9^ Cancer Epidemiology Division Cancer Council Victoria Melbourne Victoria Australia; ^10^ Department of Computational Biology St Jude Children's Research Hospital Memphis Tennessee USA; ^11^ Laboratory of Translational Genomics, Division of Cancer Epidemiology and Genetics National Cancer Institute Bethesda Maryland USA; ^12^ Quantitative Health Sciences Mayo Clinic Rochester Minnesota USA; ^13^ Department of Population Health Sciences Institute for Risk Assessment Sciences, Utrecht University Utrecht The Netherlands; ^14^ Julius Center for Health Sciences and Primary Care University Medical Center Utrecht Utrecht The Netherlands; ^15^ Surveillance and Evaluation Research Program The Kirby Institute, University of New South Wales Sydney New South Wales Australia; ^16^ Department of Medicine, Solna Karolinska Institutet Stockholm Sweden; ^17^ Hematology Center Karolinska University Hospital Stockholm Sweden; ^18^ Cancer Control Research BC Cancer Vancouver British Columbia Canada; ^19^ School of Population and Public Health University of British Columbia Vancouver British Columbia Canada; ^20^ Department of Medicine Memorial Sloan Kettering Cancer Center New York New York USA; ^21^ Centre for Epidemiology and Biostatistics Melbourne School of Population and Global Health, University of Melbourne Melbourne Victoria Australia; ^22^ Precision Medicine School of Clinical Sciences at Monash Health, Monash University Clayton Victoria Australia; ^23^ Department of Internal Medicine Carver College of Medicine, The University of Iowa Iowa City Iowa USA; ^24^ Department of Obstetrics and Gynecology New York University School of Medicine New York New York USA; ^25^ Department of Environmental Medicine New York University School of Medicine New York New York USA; ^26^ Perlmutter Cancer Center NYU Langone Medical Center New York New York USA; ^27^ Center for Chronic Immunodeficiency Institute for Immunodeficiency, University Medical Center Freiburg Freiburg Baden‐Württemberg Germany; ^28^ Department of Epidemiology and Biostatistics University of California San Francisco San Francisco California USA; ^29^ Department of Internal Medicine and Huntsman Cancer Institute University of Utah School of Medicine Salt Lake City Utah USA; ^30^ Division of Hematology‐Oncology, Department of Medicine University of California Irvine Orange California USA; ^31^ Department of Epidemiology Research, Division of Health Surveillance and Research Statens Serum Institut Copenhagen Denmark; ^32^ Department of Clinical Medicine University of Copenhagen Copenhagen Denmark; ^33^ Department of Haematology Rigshospitalet Copenhagen Denmark; ^34^ Danish Cancer Society Research Center Danish Cancer Society Copenhagen Denmark; ^35^ Channing Division of Network Medicine, Department of Medicine Brigham and Women's Hospital and Harvard Medical School Boston Massachusetts USA; ^36^ Department of Epidemiology Harvard T H Chan School of Public Health Boston Massachusetts USA; ^37^ Department of Medical Epidemiology and Biostatistics Karolinska Institutet Stockholm Sweden; ^38^ Institute of Health and Society, Clinical Effectiveness Research Group University of Oslo Oslo Norway; ^39^ Division of Cancer Epidemiology German Cancer Research Center (DKFZ) Heidelberg Baden‐Württemberg Germany; ^40^ Cancer Epidemiology Research Programme Catalan Institute of Oncology‐IDIBELL Barcelona Spain; ^41^ Unidad de Infecciones y Cáncer CIBER de Epidemiología y Salud Pública (CIBERESP) Barcelona Spain; ^42^ Regional Laboratory for Cancer Prevention Institute for Cancer Research, Prevention and Oncological Network (ISPRO) Florence Italy; ^43^ Stony Brook Cancer Center Stony Brook University Stony Brook New York USA; ^44^ Department of Medical and Surgical Sciences University of Bologna Bologna Italy; ^45^ Genomic Epidemiology Branch International Agency for Research on Cancer (IARC) Lyon France; ^46^ Genome Sciences Centre BC Cancer Vancouver British Columbia Canada; ^47^ Department of Biomedical Physiology and Kinesiology Simon Fraser University Burnaby British Columbia Canada; ^48^ Genomic Epidemiology Group German Cancer Research Center (DKFZ) Heidelberg Germany; ^49^ National Registry of Childhood Cancers APHP, CHU Paul Brousse, Villejuif and CHU de Nancy France; ^50^ Bill Lyons Informatics Centre UCL Cancer Institute, University College London London UK; ^51^ Cancer Research Center of Lyon INSERM U1052, Centre Léon Bérard Lyon France; ^52^ Department of Cancer Epidemiology and Genetics Masaryk Memorial Cancer Institute Brno Czech Republic; ^53^ Department of Hematology Hospices Civils de Lyon, Lyon Sud Hospital Pierre Benite France; ^54^ CIRI, Centre International de Recherche en Infectiologie, Team Lymphoma Immuno‐Biology Univ Lyon, Inserm, U1111, Université Claude Bernard Lyon 1, CNRS, UMR5308, ENS de Lyon Lyon France; ^55^ Department of Immunology, Genetics and Pathology Uppsala University Uppsala Sweden; ^56^ Department of Internal Medicine Mayo Clinic Rochester Minnesota USA; ^57^ INSERM U1231, EA 4184, Registre des Hémopathies Malignes de Côte d'Or University of Burgundy and Dijon University Hospital Dijon France; ^58^ Medicine and Health Sciences Department Jebsen Center for Genetic Epidemiology, NTNU Trondheim Norway; ^59^ Danish Cancer Institute Danish Cancer Society Copenhagen Denmark; ^60^ Department of Genetics Stanford University Medical School Stanford California USA; ^61^ Environmental and Occupational Epidemiology Unit Cancer Prevention and Research Institute (ISPO) Florence Italy; ^62^ Department of Pathology APHP, Necker and Robert Debré, Université Paris Cité, Institut Imagine, INSERM U1163 Paris France; ^63^ Department of Epidemiology University of North Carolina at Chapel Hill Chapel Hill North Carolina USA; ^64^ Carolina Center for Genome Sciences University of North Carolina at Chapel Hill Chapel Hill North Carolina USA; ^65^ Unit of Medical Statistics and Epidemiology, Department Translational Medicine University of Eastern Piedmont “Amedeo Avogadro” Novara Italy; ^66^ Cancer Epidemiology and Prevention Research Unit School of Public Health, Imperial College London London UK; ^67^ Department of Family Medicine and Public Health Sciences Wayne State University Detroit Michigan USA; ^68^ Department of Clinical Pathology Melbourne Medical School, The University of Melbourne Melbourne Victoria Australia; ^69^ School of Nursing, Psychotherapy and Community Health Dublin City University Dublin Ireland; ^70^ Division of Public Health Sciences Fred Hutchinson Cancer Research Center Seattle Washington, DC USA; ^71^ Cancer Epidemiology Unit University of Oxford Oxford UK; ^72^ Division of Health Analytics City of Hope Beckman Research Institute Duarte California USA; ^73^ Department of Epidemiology Brown University Providence Rhode Island USA

## Abstract

Obesity has been associated with non‐Hodgkin lymphoma (NHL), but the evidence is inconclusive. We examined the association between genetically determined adiposity and four common NHL subtypes: diffuse large B‐cell lymphoma (DLBCL), follicular lymphoma, chronic lymphocytic leukemia, and marginal zone lymphoma, using eight genome‐wide association studies of European ancestry (*N* = 10,629 cases, 9505 controls) and constructing polygenic scores for body mass index (BMI), waist‐to‐hip ratio (WHR), and waist‐to‐hip ratio adjusted for BMI (WHRadjBMI). Higher genetically determined BMI was associated with an increased risk of DLBCL [odds ratio (OR) per standard deviation (SD) = 1.18, 95% confidence interval (95% CI): 1.05–1.33, *p* = .005]. This finding was consistent with Mendelian randomization analyses, which demonstrated a similar increased risk of DLBCL with higher genetically determined BMI (OR_per SD_ = 1.12, 95% CI: 1.02–1.23, *p* = .03). No significant associations were observed with other NHL subtypes. Our study demonstrates a positive link between a genetically determined BMI and an increased risk of DLBCL, providing additional support for increased adiposity as a risk factor for DLBCL.

## Graphical abstract

What's new?

Obesity has been linked to the risk of non‐Hodgkin lymphoma (NHL), but the evidence remains inconclusive. Here, the authors investigate the possibility of shared genetic risk factors between obesity and four common NHL types. Drawing on data from 8 genome‐wide association studies, they found that a higher genetically determined BMI was associated with a higher risk of just one NHL type, diffuse large B cell lymphoma (DLBCL). This positive association was confirmed using Mendelian randomization.

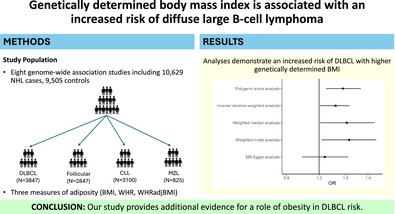

**Abbreviations**
BMI: body mass index
CLL: chronic lymphocytic leukemia
DLBCL: diffuse large B‐cell lymphoma
FL: follicular lymphoma
IARC: International Agency for Research on Cancer
MZL: marginal zone lymphoma
NHL: non‐Hodgkin lymphoma
OR: odds ratio
PGS: polygenic score
SNP: single nucleotide polymorphism
WHR: waist‐to‐hip ratio
WHRadjBMI: waist‐to‐hip ratio adjusted for BMI
95% CI: 95% confidence interval

## 1 INTRODUCTION

Excess adiposity, possibly through mechanisms involving chronic inflammation, is a suggested risk factor for B‐cell lymphomas. Although the findings are not entirely consistent,^1^, ^2^, ^3^ several observational studies of non‐Hodgkin lymphoma have shown an association between obesity and lymphoma risk.^4^, ^5^, ^6^, ^7^, ^8^ Of more common lymphoma subtypes, the evidence is most convincing for diffuse large B‐cell lymphoma (DLBCL), where excess weight‐for‐height in later adulthood has been found to increase the risk of disease.^2^, ^4^, ^5^, ^6^, ^7^, ^9^, ^10^ There is also some evidence that being overweight or obese in young adulthood may contribute to DLBCL risk.^10^, ^11^, ^12^, ^13^, ^14^ The evidence is less clear for other B‐cell lymphomas, such as follicular lymphoma (FL), chronic lymphocytic leukemia (CLL), and marginal zone lymphoma (MZL).^5^, ^6^, ^15^ These lymphomas are more indolent in nature, and weight loss in the years leading up to diagnosis could have attenuated any observed association of later adult obesity with disease etiology. Although no associations were observed with body mass index (BMI) and risk of CLL or MZL,^15^, ^16^ the InterLymph Consortium reported an association between higher BMI in young adulthood and increased risk of FL in a large pooled analysis.^17^

To date, the majority of epidemiologic studies of lymphoma have used BMI to estimate adiposity, largely due to the ease of obtaining height and weight information, but other measures, such as waist‐to‐hip ratio (WHR), may provide better indicators of body fat distribution. A larger WHR is indicative of greater abdominal, rather than gluteal, fat deposition, and has been associated with an increased risk of type 2 diabetes, cardiovascular disease, and some cancers.^18^, ^19^, ^20^ The relationship between WHR and NHL has been studied less often. Although most studies have not observed an association with WHR,^2^, ^13^, ^21^, ^22^, ^23^, ^24^ these studies have generally been small and underpowered to detect associations with specific NHL subtypes. A meta‐analysis suggested that higher WHR may be associated with DLBCL, but not FL.^14^ Some of the observed discrepancies could be due to the known difficulties in studying anthropometry. These include the reliability of height and weight when self‐reported, as is common in studies of lymphoma; the timeframe of anthropometric information relative to lymphoma diagnosis, especially when collected retrospectively; and the different ages, time periods, and places of study where the population prevalence of obesity may vary. Changes in body composition over an individual's lifetime complicate the study of the relationships between obesity and disease risk.

Although adult adiposity is a marker of multiple biological influences reflecting the interplay of genetics and environment, positive associations between adiposity and NHL risk raise the possibility of shared genetic risk factors. Genetic alleles are randomly allocated at conception, not affected by disease status or environmental exposures, measured reliably, and unchanged over the life course of an individual. Hundreds of genetic variants have been identified to be associated with measures of adiposity, BMI, WHR, and WHR adjusted for BMI (WHRadjBMI); the variants for BMI and WHRadjBMI explain 6.0% and 3.9% of the variance, respectively.^25^, ^26^ Many of the loci identified for WHR without adjustment for BMI overlap those loci identified for BMI, making interpretation of the results difficult as associations observed for WHR may be due to correlation with BMI. In contrast, the loci identified for WHRadjBMI are distinct from and independent of those identified for BMI and reflect different biological pathways. By combining SNP‐specific associations with adiposity into a single measure, a polygenic score (PGS),^27^ one can examine the association between genetically determined adiposity and NHL risk while circumventing some of the aforementioned methodological complexities for characterizing body size. Mendelian randomization, an analytic approach using genetic variation, can be used to investigate potentially causal relationships between risk factors, such as obesity, and disease risk.^28^ Indeed, previous Mendelian randomization studies have used adiposity‐related polygenic scores as instruments to further elucidate and characterize the etiology of obesity‐related diseases.^29^, ^30^

Here, we examined the association between genetically determined adiposity and NHL risk using data from eight GWAS, including 10,629 NHL cases and 9505 controls. As evidence suggests that subtypes of NHL have distinctly different etiologies,^31^, ^32^ we focused our analysis on the risk of four major NHL subtypes, DLBCL, FL, CLL, and MZL, and investigated associations with BMI, WHR, and WHRadjBMI.

## 2 MATERIALS AND METHODS

We used previously genotyped samples of European ancestry and imputed data from eight GWAS of NHL (Supplementary Table 1). Full details of participating studies, including detailed information on quality control and data cleaning, have been previously reported for DLBCL, FL, CLL, and MZL.^33^, ^34^, ^35^, ^36^ Briefly, the largest GWAS, the InterLymph NHL GWAS, was a pooled study consisting of NHL cases and controls from 22 studies of NHL: nine prospective cohort studies, eight population‐based case–control studies, and five hospital or clinic‐based case–control or case‐series studies. The other seven GWAS were the University of California at San Francisco Molecular Epidemiology of Non‐Hodgkin Lymphoma study (UCSF2),^37^ University of California at San Francisco Molecular Epidemiology of Non‐Hodgkin Lymphoma study (UCSF1)/Nurses' Health Study (NHS),^38^ Scandinavian Lymphoma Etiology Study (SCALE),^39^ Genetic Epidemiology of CLL Consortium (GEC),^40^ Groupe d'Etude des Lymphomes de l'Adulte (GELA)/European Prospective Investigation into Cancer, Chronic Diseases, Nutrition, and Lifestyles (EPIC),^41^, ^42^ Mayo Clinic Case–Control Study of Diffuse Large B‐cell Lymphoma (Mayo‐DLBCL),^43^ and the Utah Chronic Lymphocytic Leukemia Study (Utah). Genotyping was performed on commercially available Illumina and Affymetrix platforms (Supplementary Table 1), and standard quality control and filtering metrics (i.e., low SNP and sample call rates, sex discordance, deviation from Hardy–Weinberg proportions) were applied to each GWAS. Imputation was performed separately for each of the eight GWAS using IMPUTE2^44^ and the 1000 Genomes Project reference panel.^45^ Across the eight GWAS, genotype data were available for a total of 10,629 NHL cases, including 3100 CLL, 3857 DLBCL, 2847 FL, and 825 MZL cases, and up to 9505 controls.

To evaluate the genetically determined adiposity and the risk of NHL, we constructed separate PGS for BMI, WHR, and WHRadjBMI using published genetic loci from the Genetic Investigation of ANthropometric Traits (GIANT) Consortium, which included 941 independent SNPs associated with BMI, 382 independent SNPs associated with WHR, and 463 independent SNPs associated with WHRadjBMI.^25^, ^26^ Two SNPs for BMI, 4 SNPs for WHR, and nine SNPs for WHRadjBMI were not available in our GWAS and were excluded. After Steiger filtering,^46^ 917 SNPs remained for BMI, 358 SNPs for WHR, and 430 SNPs for WHRadjBMI. The polygenic scores were computed for each individual (*i*) as the sum of the allelic dosage for each SNP multiplied by the reported weight of the association with BMI, WHR, or WHRadjBMI, as shown below:

$$\text{PG}S_{i}=\sum_{j=1}^{k}w_{j}x_{\mathrm{ij}}$$

where *w*_*j*_ is the weight or beta coefficient for the *j*th SNP derived from the literature and *x*_*ij*_ is the allelic dosage of the *j*th SNP. In our study, we used the previously published per‐risk‐allele *β*‐estimates for the European sex‐combined meta‐analysis for BMI, WHR, or WHRadjBMI.^25^, ^26^

As previous studies have demonstrated substantial etiologic heterogeneity among NHL subtypes,^31^, ^32^ we analyzed each of the four NHL subtypes in this study separately. Logistic regression was used to estimate odds ratios (ORs) and 95% confidence intervals (95% CI) for the association with genetically determined BMI, WHR, and WHRadjBMI, separately by GWAS and by subtype. Each PGS was modeled as a continuous variable and categorized into quartiles with quartile cutoffs defined by the distribution among controls. Models were adjusted for sex, age at NHL diagnosis or control selection, and GWAS‐specific statistically significant (*p* <.05) principal components for population stratification. The regression model for the UCSF1/NHS study was not adjusted for sex, as all controls were female. As previous studies have demonstrated sex differences for waist‐related traits,^26^ as secondary analyses, we also performed sex‐stratified analyses using: (1) the same weights for the PGS as the sex‐combined analysis, which allowed us to test for differences in the effect size estimates using a Wald test, and (2) sex‐specific PGS, which were constructed using the SNPs and weights previously identified for each sex.^25^, ^26^ All analyses were adjusted for age and study‐specific statistically significant principal components. The UCSF1/NHS study was excluded from sex‐specific analyses, as all controls were women. Fixed‐effects meta‐analysis was used to combine association results from the included GWAS for each NHL subtype. Between‐study heterogeneity was evaluated by Cochran's *Q* test and was quantified using the *I* ^*2*^ metric. The meta‐analysis was conducted using the package *metan* in Stata v16 (StataCorp, College Station, TX). The *p*‐values for between‐sex heterogeneity were calculated using a Wald test. A *p*‐value <.05 was considered nominally statistically significant, and *p*‐values of 0.05–0.10 were considered suggestive. To account for multiple testing and control for the false discovery rate, *q*‐values were calculated using Benjamini‐Hochberg, which is less conservative than Bonferroni.^47^

In addition, we conducted a Mendelian Randomization analysis to assess the potential causal relationship using the inverse‐variance weighted (IVW), MR‐Egger, weighted median, and weighted mode methods as implemented in the *TwoSampleMR* R package.^48^ We focused on the IVW approach as our primary method for analysis. Fixed effects models were used for the IVW analysis. The same SNPs were used for the Mendelian analysis as for the PGS analysis. Using only summary‐level data and under certain strict assumptions,^28^ the exponentiated coefficient calculated in this manner can be interpreted as the causal OR of a given NHL subtype associated with a one‐unit increase in the adiposity trait. The Mendelian randomization analysis requires several assumptions: (1) PGS is related to the exposure, (2) the PGS is not related to other exposures that could be confounders, and (3) the PGS affects the outcome only through the exposure. There should be no directional or horizontal pleiotropy. To assess the potential presence of directional pleiotropic effects, we performed Egger regression and estimated the heterogeneity of SNP association using Cochrane's *Q*‐statistic. Scatter plots were used to visualize the associations, and leave‐one‐out analyses were used to evaluate the impact of outlier SNPs.

## 3 RESULTS

**FIGURE 1:**
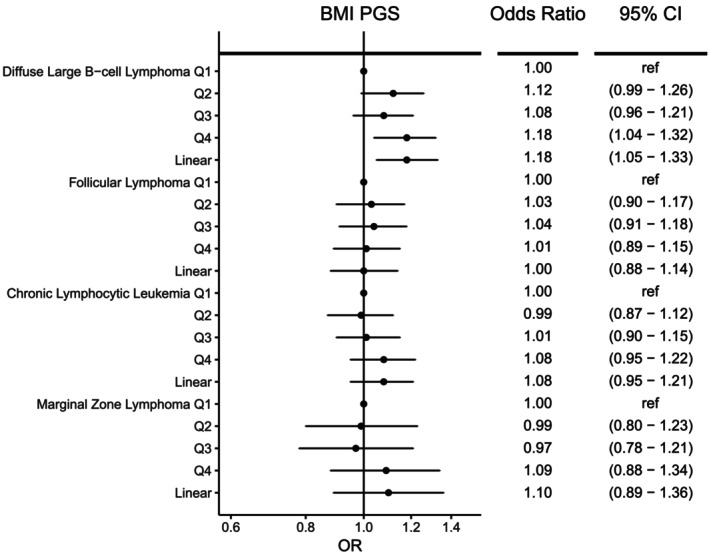
Risk of four non‐Hodgkin lymphoma subtypes associated with genetically determined body mass index (BMI). Odds ratios (ORs) and 95% confidence intervals (CIs) are shown for quartiles (Q1‐Q4) of the BMI polygenic score (PGS) and BMI PGS modeled as a linear term.

Overall, higher genetically determined BMI was associated with an elevated risk of DLBCL (OR_per SD_ = 1.18, 95% CI = 1.05–1.33, *p* = .005) (Figure 1), which remained significant after adjustment for multiple testing (*q* = 0.02). Compared to the lowest quartile, those in the highest quartile of genetically determined BMI had an 18% increased risk of DLBCL (OR = 1.18, 95% CI:1.04–1.32). After stratifying by sex (Supplementary Table 2), we observed a positive association with increasing BMI PGS and risk of DLBCL for men (OR_per SD_ = 1.23, 95% CI 1.05–1.44, *p* = .01) with weaker evidence of an association in women (OR_per SD_ = 1.10, 95% CI 0.92–1.31, *p* = .29); however, the difference was not statistically significant (p_heterogeneity_ = 0.35). Further analyses using sex‐specific PGS for BMI yielded similar positive associations for both men and women (Supplementary Table 3). Overall, there was no association between genetically predicted BMI and the risk of FL, CLL, or MZL (Figure 1). After stratifying by sex (Supplementary Table 2), higher genetically determined BMI was suggestively associated with an increased risk of CLL among men (OR_per SD_ = 1.17, 95% CI: 1.00–1.37, *p* = .05) with men in the highest quartile displaying a 15% increased risk of CLL compared to the lowest quartile of BMI PGS (OR = 1.15, 95% CI: 0.98–1.34). No association was observed for women (OR_per SD_ = 0.97, 95% CI: 0.79–1.18, *p* = .73), and there was no significant heterogeneity by sex for CLL risk (p_heterogeneity_ = 0.14). Analyses using sex‐specific PGS showed a significant positive association for genetically predicted BMI among men but not women (Supplementary Table 3). Little evidence of heterogeneity among GWAS was observed for the BMI PGS (P_heterogeneity_ >0.05, Supplementary Table 4).

**FIGURE 2:**
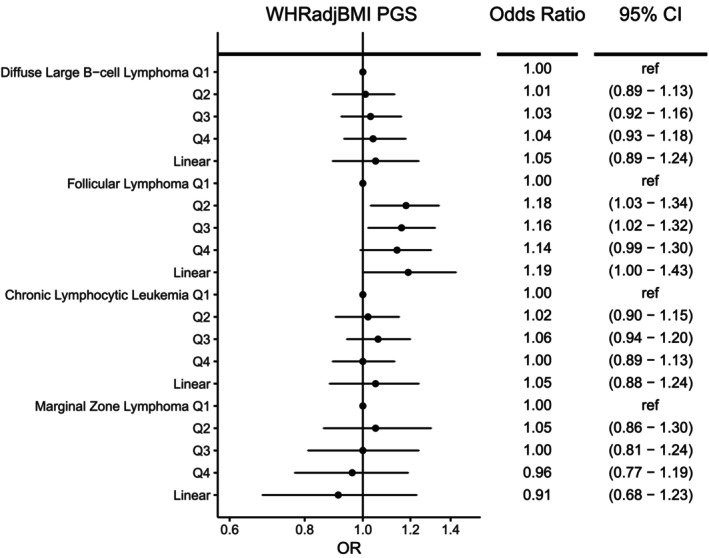
Risk of four non‐Hodgkin lymphoma subtypes associated with genetically determined waist‐to‐hip ratio adjusted for BMI (WHRadjBMI). Odds ratios (ORs) and 95% confidence intervals (CIs) are shown for quartiles (Q1‐Q4) of the WHRadjBMI polygenic score (PGS) and WHRadjBMI PGS modeled as a linear term.

**FIGURE 3:**
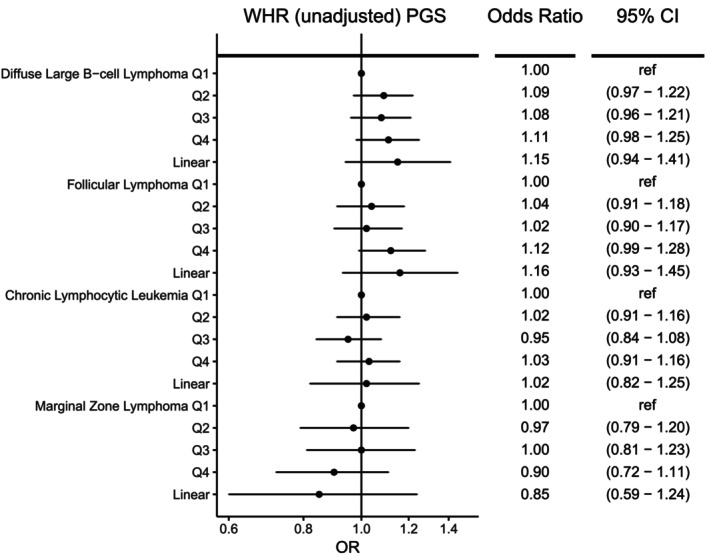
Risk of four non‐Hodgkin lymphoma subtypes associated with genetically determined waist‐to‐hip ratio (WHR). Odds ratios (ORs) and 95% confidence intervals (CIs) are shown for quartiles (Q1‐Q4) of the WHR polygenic score (PGS) and WHR PGS modeled as a linear term.

For men and women combined, no significant associations were observed for any NHL subtype with genetically determined body fat distribution as measured by WHR or WHRadjBMI PGS (Figures 2 and 3), although there was a suggestive association with higher WHRadjBMI and increased FL risk (OR = 1.19 [1.00–1.43], *p* = 0.06). In sex‐specific analyses (Supplementary Tables 5), although not significant after adjustment for multiple testing (*q* = 0.18), higher WHRadjBMI PGS was nominally associated with lower risk of MZL for men (OR_per SD_ = 0.60, 95% CI = 0.38–0.94, *p* = .03) but not women (OR_per SD_ = 1.25, 95% CI = 0.84–1.88, *p* = .28), with evidence of heterogeneity between the sexes (p_heterogeneity_ = 0.02). Men in the highest quartile of WHRadjBMI PGS had a 33% reduction in the risk of MZL compared to the lowest quartile (OR = 0.67, 95% CI: 0.49–0.93). Additional adjustment for genetically determined BMI in the WHRadjBMI analyses did not substantially alter the ORs observed (data not shown). Consistent with the results for WHRadjBMI, a suggestive reduction in MZL risk was also observed for men with higher genetically predicted WHR (OR_per SD_ = 0.58 [0.34–1.02], *p* = .06), but not women (OR_per SD_ = 1.13 [0.68–1.86], *p* = .64) (P_heterogeneity_ = 0.08, Supplementary Table 6). Analyses using sex‐specific PGS showed a suggestive reduction in MZL risk for WHRadjBMI among men (Supplementary Tables 7 and 8). No significant linear trends were observed with other subtypes in sex‐specific analyses. With the exception of a few individual quartiles, there was little evidence for heterogeneity among the individual GWAS (Supplementary Tables 9 and 10).

**FIGURE 4:**
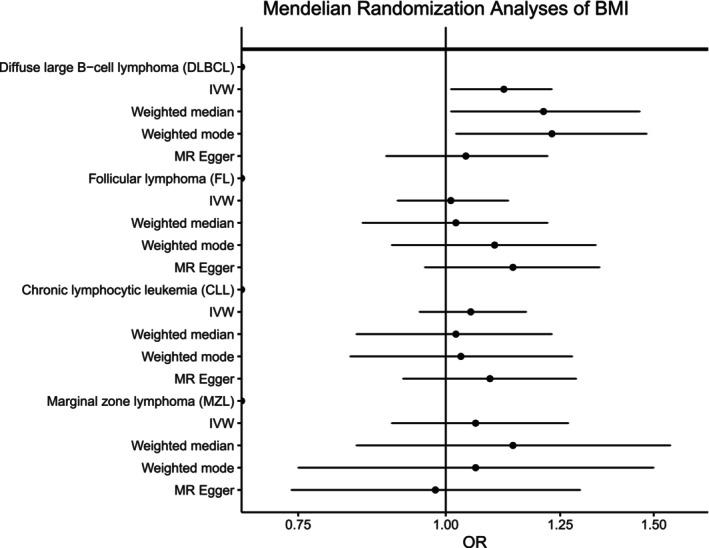
Mendelian randomization analyses of body mass index (BMI) and the risk of four non‐Hodgkin lymphoma subtypes. Odds ratios (ORs) and 95% confidence intervals (CIs) are shown for each subtype using the inverse variance weighted (IVW), weighted median, weighted mode, and MR Egger methods.

**FIGURE 5:**
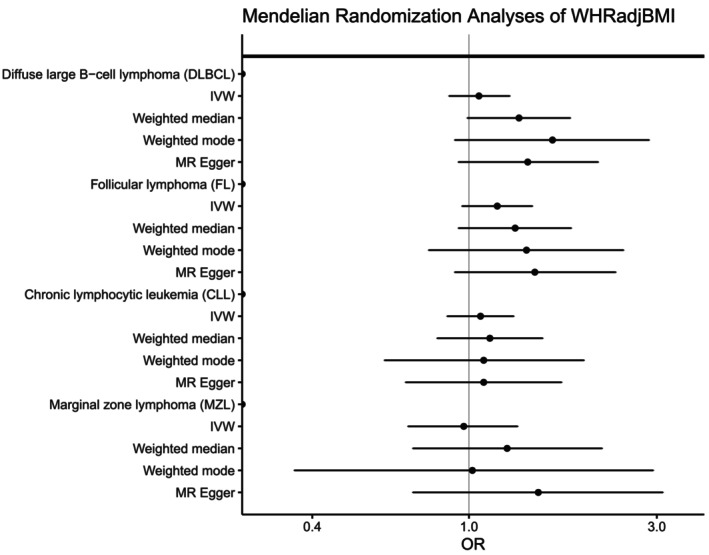
Mendelian randomization analyses of waist‐to‐hip ratio adjusted for body mass index (WHRadjBMI) and the risk of four non‐Hodgkin lymphoma subtypes. Odds ratios (ORs) and 95% confidence intervals (CIs) are shown for each subtype using the inverse variance weighted (IVW), weighted median, weighted mode, and MR Egger methods.

**FIGURE 6:**
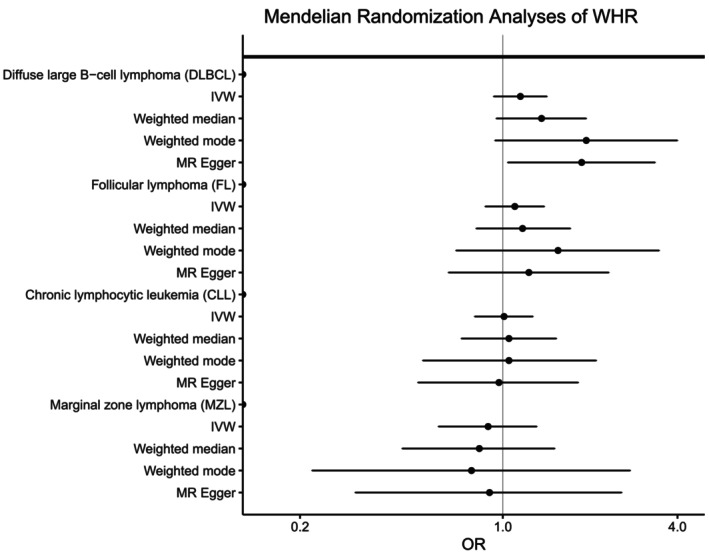
Mendelian randomization analyses of waist‐to‐hip ratio (WHR) and the risk of four non‐Hodgkin lymphoma subtypes. Odds ratios (ORs) and 95% confidence intervals (CIs) are shown for each subtype using the inverse variance weighted (IVW), weighted median, weighted mode, and MR Egger methods.

To further explore the relationship between adiposity and NHL risk, we conducted Mendelian randomization analyses; we discovered generally high concordance between the associations calculated using individual‐level PGS with those estimated using the inverse variance‐weighted (IVW), weighted median, and weighted mode methods and summary‐level statistics for BMI, WHR, and WHRadjBMI for all NHL subtypes (Figures 4, 5, 6, Supplementary Table 11). Similar to what we observed with the individual‐level PGS, higher genetically determined BMI was associated with an increased risk of DLBCL using the IVW method (OR_per SD_ = 1.12, 95% CI: 1.02–1.23, *p* = .03) with similar results for the weighted median and weighted mode. No significant associations were observed for the other NHL subtypes. Egger regression did not detect evidence of overall bias in the IVW estimate due to directional pleiotropy in any of the analyses (*p* >.05 for all, Supplementary Table 11). Scatterplots showing SNP‐specific associations for BMI, WHR, and WHRadjBMI with each NHL subtype are shown in Supplementary Figures 1–3; no individual SNPs were associated with any NHL subtype at a Bonferroni significance level of 5.5 × 10^−5^ for BMI, 1.4 × 10^−4^ for WHR, and 1.2 × 10^−4^ for WHRadjBMI. There was little evidence of heterogeneity in SNP effects (*p*>.01 for all, Supplementary Table 11) Leave‐one‐SNP‐out analyses yielded similar results and did not provide evidence of any SNP unduly influencing the results (data not shown).

## 4 DISCUSSION

In this large study of genetically determined adiposity and NHL risk, we observed a positive association between genetically determined BMI and risk of DLBCL. The International Agency for Research on Cancer (IARC) previously concluded that there was limited, but insufficient, evidence for an association between excess body fatness and DLBCL.^49^ Our study provides additional evidence for a role of adiposity in DLBCL risk. Our results are consistent with epidemiologic studies of measured or self‐reported BMI, including a pooled analysis of prospective cohorts that reported a statistically significant positive association between BMI and risk of DLBCL^10^ and a pooled analysis based on case–control data from the InterLymph Consortium that discovered elevated risks for DLBCL with increasing young adult BMI.^50^ Prospective data from the Nurses' Health Study and Health Professionals Follow‐up Study and other studies further support a role for BMI throughout the life course.^11^, ^12^, ^13^, ^14^

A positive association between BMI and DLBCL risk was further supported by our Mendelian randomization analysis, suggesting a potential causal relationship between BMI and DLBCL risk. Obesity has been linked to many altered metabolic processes, including alterations in the insulin‐like growth factor pathway, sex hormones, and adipokines as well as chronic low‐grade inflammation,^51^ which may promote lymphomagenesis. However, some caution should be exercised in inferring causality from this analysis. Although the intercept term calculated using Egger regression for DLBCL was not significantly different from zero, this statistical test does not rule out the existence of directional pleiotropy. BMI is a polygenic trait, and the etiology of DLBCL has yet to be fully elucidated. The existence of biological pleiotropy at one or more loci has the potential to violate the Instrument Strength Independent of Direct Effect (InSIDE) assumption of Mendelian randomization if those variants are related to an unmeasured confounder or act by the same pleiotropic mechanism (e.g., innate immunity).

Given the epidemiologic associations between measured or reported adult BMI and risk of DLBCL in both men and women, it is intriguing that our study found a positive association between the genetically determined BMI and DLBCL risk in men, but less evidence for women. There is ample evidence for sex‐specific aspects of adiposity. For instance, women are relatively protected from certain obesity‐related diseases, such as cardiovascular disease, until menopause when the resulting hormonal changes result in a shift in fat distribution.^52^ Twin studies suggest that the genetic influence on BMI may differ between men and women,^53^ and, across multiple populations, that sex‐specific genetic influences on BMI are strongest after puberty.^54^ It is possible that overall adiposity plays a stronger role in the risk of DLBCL for men. However, it is important to note that the difference in risk between men and women was not statistically significant in our study, and the elevated risk observed for men could be a chance finding.

For genetically inferred WHRadjBMI, we discovered a nominally lower risk of MZL with higher WHRadjBMI PGS among men but not women. Although collider bias is possible, additional adjustment for genetically determined BMI in the WHRadjBMI analyses did not substantially alter the associations observed, and results were similar for WHR analyses. Although the difference between sexes was significant, some caution should be exercised in drawing inferences as this was a subgroup finding. The result was not statistically significant after adjustment for multiple testing (*q* = 0.18), and heritability of WHRadjBMI and variance explained by the reported loci is less for men compared to women.^26^ Although not statistically significant, we also observed a borderline increased risk of FL in the PGS analysis with similar, albeit weaker, evidence in the Mendelian randomization analyses. Although we used over 400 genetic variants to construct the PGS and Mendelian randomization instrument for WHRadjBMI, these variants combined explain only 1.0%–3.9% of the variation.^26^ As such, there is likely misclassification in the ranking of individuals for these traits based on their PGS. Assuming that the misclassification is non‐differential, this could have biased our observed results toward the null. Other studies have reported positive associations between increasing genetically inferred BMI and WHRadjBMI with risk of colorectal cancer using a similar approach,^29^, ^55^ indicating that it is possible to uncover associations with cancer risk with only a small percentage of the variation captured by the score. However, these studies had a larger sample size, and we may have been underpowered to detect a modest association with risk in our study. Burgess et al. reported that a sample size of ~10,000 cases is required to detect a modest association in Mendelian randomization analyses using an instrumental variable explaining 5% of the variance.^56^

Although the literature on WHR and cancer risk is limited compared with that on BMI and cancer, previous work that examined WHR as a risk factor, most of which has been conducted in women, has not found associations with NHL risk.^2^, ^21^, ^22^, ^23^, ^24^, ^57^ WHR represents a different measure of adiposity that incorporates the observed protective effects of an increased femoral‐gluteal muscle,^58^ and studies of cardiovascular disease have shown associations with WHR independent of BMI.^19^ WHRadjBMI loci have been shown to be enriched for genes expressed in adipose tissue itself, and pathway analyses implicated genes involved in angiogenesis, transcriptional regulation, and insulin resistance,^59^ the latter of which are also established pathways for carcinogenesis. However, we only found limited evidence for a role of WHR and WHRadjBMI in NHL risk in subgroup analyses in our study.

Our study has limitations that are important to consider. Our participants were sampled from populations of European descent, limiting the generalizability of results to other ethnic groups, but this also has the benefit of reducing bias from population stratification. As many of our subjects came from retrospective case–control studies, there is the possibility of participation bias, as obese subjects may be less likely to be controls; however, genotypes, distributed at birth, are unlikely to be related to study participation. We did not have individual‐level data on BMI or WHR from all studies and could not evaluate the variance explained by the PGS in our study. Although we were able to combine multiple studies of NHL together in one analysis and achieve sample sizes of several thousand cases for three out of the four subtypes, our study had limited power to detect modest associations between these anthropometric traits and specific NHL subtypes. Larger studies are needed to further explore the suggestive findings in this study. Previous studies of measured or self‐reported adiposity and cancer risk have been susceptible to recall bias, reverse causation, residual confounding, and misclassification. By utilizing genetically determined measures of adiposity, our results are less sensitive to these concerns and provide additional insight into the role of adiposity on NHL risk, though at the expense of the non‐genetic contribution.

In conclusion, our study provides evidence supporting a positive association between BMI and DLBCL risk, consistent with the previously reported epidemiologic studies of measured or self‐reported adult BMI and risk of DLBCL. Future exploration of biological pathways that underlie the association with BMI and DLBCL is warranted as well as the evaluation of BMI with DLBCL subtypes, as these may provide more insight into the etiology of NHL.

## AUTHOR CONTRIBUTIONS

**Amy Moore:** Conceptualization; data curation; formal analysis; investigation; methodology; writing – original draft; writing – review and editing. **Eleanor Kane:** Conceptualization; methodology; resources; writing – review and editing. **Lauren R. Teras:** Conceptualization; methodology; resources; writing – review and editing. **Mitchell J. Machiela:** Conceptualization; methodology; writing – review and editing. **Joshua Arias:** Conceptualization; formal analysis; writing – review and editing. **Orestis A. Panagiotou:** Conceptualization; methodology; writing – review and editing. **Alain Monnereau:** Conceptualization; methodology; resources; writing – review and editing. **Nicole Wong Doo:** Conceptualization; methodology; resources; writing – review and editing. **Zhaoming Wang:** Conceptualization; formal analysis; methodology; writing – review and editing. **Susan L. Slager:** Resources; writing – review and editing. **Roel C. H. Vermeulen:** Resources; writing – review and editing. **Claire M. Vajdic:** Resources; writing – review and editing. **Karin E. Smedby:** Resources; writing – review and editing. **John J. Spinelli:** Resources; writing – review and editing. **Joseph Vijai:** Resources; writing – review and editing. **Graham G. Giles:** Resources; writing – review and editing. **Brian K. Link:** Resources; writing – review and editing. **Alan A. Arslan:** Resources; writing – review and editing. **Alexandra Nieters:** Resources; writing – review and editing. **Paige M. Bracci:** Resources; writing – review and editing. **Nicola J. Camp:** Resources; writing – review and editing. **Gilles Salles:** Resources; writing – review and editing. **Wendy Cozen:** Resources; writing – review and editing. **Henrik Hjalgrim:** Resources; writing – review and editing. **Immaculata De Vivo:** Resources; writing – review and editing. **Hans‐Olov Adami:** Resources; writing – review and editing. **Demetrius Albanes:** Resources; writing – review and editing. **Nikolaus Becker:** Resources; writing – review and editing. **Yolanda Benavente:** Resources; writing – review and editing. **Simonetta Bisanzi:** Resources; writing – review and editing. **Paolo Boffetta:** Resources; writing – review and editing. **Paul Brennan:** Resources; writing – review and editing. **Angela R. Brooks‐Wilson:** Resources; writing – review and editing. **Federico Canzian:** Resources; writing – review and editing. **Jacqueline Clavel:** Resources; writing – review and editing. **Lucia Conde:** Resources; writing – review and editing. **David G. Cox:** Resources; writing – review and editing. **Karen Curtin:** Resources; writing – review and editing. **Lenka Foretova:** Resources; writing – review and editing. **Hervé Ghesquières:** Resources; writing – review and editing. **Bengt Glimelius:** Resources; writing – review and editing. **Thomas M. Habermann:** Resources; writing – review and editing. **Jonathan N. Hofmann:** Resources; writing – review and editing. **Qing Lan:** Resources; writing – review and editing. **Mark Liebow:** Resources; writing – review and editing. **Anne Lincoln:** Resources; writing – review and editing. **Marc Maynadie:** Resources; writing – review and editing. **James McKay:** Resources; writing – review and editing. **Mads Melbye:** Resources; writing – review and editing. **Lucia Miligi:** Resources; writing – review and editing. **Roger L. Milne:** Resources; writing – review and editing. **Thierry J. Molina:** Resources; writing – review and editing. **Lindsay M. Morton:** Resources; writing – review and editing. **Kari E. North:** Resources; writing – review and editing. **Kenneth Offit:** Resources; writing – review and editing. **Marina Padoan:** Resources; writing – review and editing. **Sara Piro:** Resources; writing – review and editing. **Alpa V. Patel:** Resources; writing – review and editing. **Mark P. Purdue:** Resources; writing – review and editing. **Vignesh Ravichandran:** Resources; writing – review and editing. **Elio Riboli:** Resources; writing – review and editing. **Richard K. Severson:** Resources; writing – review and editing. **Melissa C. Southey:** Resources; writing – review and editing. **Anthony Staines:** Resources; writing – review and editing. **Lesley F. Tinker:** Resources; writing – review and editing. **Ruth C. Travis:** Resources; writing – review and editing. **Sophia S. Wang:** Resources; writing – review and editing. **Elisabete Weiderpass:** Resources; writing – review and editing. **Stephanie Weinstein:** Resources; writing – review and editing. **Tongzhang Zheng:** Resources; writing – review and editing. **Stephen J. Chanock:** Resources; writing – review and editing. **Nathaniel Rothman:** Resources; writing – review and editing. **Brenda M. Birmann:** Resources; writing – review and editing. **James R. Cerhan:** Conceptualization; methodology; resources; writing – review and editing. **Sonja I. Berndt:** Conceptualization; data curation; formal analysis; funding acquisition; investigation; methodology; resources; supervision; writing – original draft; writing – review and editing.

## CONFLICT OF INTEREST STATEMENT

G. Salles is on the advisory board for Abbvie, Beigene, BMS, Genentech/Roche, Genmab, Incyte, Ipsen, Janssen, Kite/Gilead, Loxo/Lilly, Merck, Novartis, and Nurix; consults for Abbvie, Atbtherapeutics, Beigene, BMS, Debiopharm, Genentech/Roche, Genmab, Innate Pharma, Incyte, Ipsen, Kite/Gilead, Modex, Molecular Partners, Nordic, Nanovector, Orna Therapeutics, and Treeline; receives research support from Abbvie, Genentech, Genmab, Janssen, Ipsen, and Nurix; and is a shareholder of Owkin. T.M. Habermann is on the data monitoring committee for Seagen and Eli Lilly and receives research program support from Roche/Genentech, BMS, and Genmab. The remaining authors have no conflicts of interest to declare.

## ETHICS STATEMENT

The study was conducted according to the guidelines of the Declaration of Helsinki, and approved by the following Institutional Review Boards: ATBC:(NCI Special Studies Institutional Review Board), BCCA: UBC BC Cancer Research Ethics Board, CPS‐II: American Cancer Society, ELCCS: Northern and Yorkshire Research Ethics Committee, ENGELA: IRB00003888—Comite d’ Evaluation Ethique de l'Inserm IRB # 1, EPIC: Imperial College London, EpiLymph: International Agency for Research on Cancer, HPFS: Harvard School of Public Health (HSPH) Institutional Review Board, Iowa‐Mayo SPORE: University of Iowa Institutional Review Board, Italian GxE: Comitato Etico Azienda Ospedaliero Universitaria di Cagliari, Mayo Clinic Case–Control: Mayo Clinic Institutional Review Board, MCCS: Cancer Council Victoria's Human Research Ethics Committee, MD Anderson: University of Texas MD Anderson Cancer Center Institutional Review Board, MSKCC: Memorial Sloan‐Kettering Cancer Center Institutional Review Board, NCI‐SEER (NCI Special Studies Institutional Review Board), NHS: Partners Human Research Committee, Brigham and Women's Hospital, NSW: NSW Cancer Council Ethics Committee, NYU‐WHS: New York University School of Medicine Institutional Review Board, PLCO: (NCI Special Studies Institutional Review Board), SCALE: Scientific Ethics Committee for the Capital Region of Denmark, SCALE: Regional Ethical Review Board in Stockholm (Section 4) IRB#5, UCSF2: University of California San Francisco Committee on Human Research, WHI: Fred Hutchinson Cancer Research Center, Yale: Human Investigation Committee, Yale University School of Medicine. Informed consent was obtained from all subjects involved in the study.

## Supporting information


**Data S1:** Supporting information

## Data Availability

Genotype data from the NCI NHL GWAS is available on dbGaP (phs000801.v2.p1) for research purposes in accordance with dbGaP data access policies. Other data that support the findings of this study are available for research purposes through the InterLymph Consortium upon approval in accordance with institutional review boards and general data protection regulations.
