# Supplementary material for: Genetically determined body mass index is associated with diffuse large B‐cell lymphoma in polygenic and Mendelian randomization analyses
Source: Int J Cancer. 2025 Sep 5;158(1):45–59. doi: 10.1002/ijc.70039 (PMC12588556; doi:10.1002/ijc.70039)
Supplement: Supplementary file 1 — Data S1: Supporting information [file IJC-158-45-s001.pdf]

Genetically determined body mass index is associated with diffuse large B-cell lymphoma in polygenic and Mendelian randomization analyses

Amy Moore, Eleanor Kane, Lauren R Teras, Mitchell J Machiela, Joshua Arias, Orestis A Panagiotou, Alain Monnereau, Nicole Wong Doo, Zhaoming Wang, Susan L Slager, Roel C H Vermeulen, Claire M Vajdic, Karin E Smedby[,], John J Spinelli[,], Joseph Vijai, Graham G Giles, Brian K Link, Alan A Arslan, Alexandra Nieters, Paige M Bracci, Nicola J Camp, Gilles Salles, Wendy Cozen, Henrik Hjalgrim, Immaculata De Vivo, Hans-Olov Adami[,,], Demetrius Albanes, Nikolaus Becker, Yolanda Benavente, Simonetta Bisanzi, Paolo Boffetta[,], Paul Brennan, Angela R Brooks-Wilson[,], Federico Canzian, Jacqueline Clavel, Lucia Conde, David G Cox, Karen Curtin, Lenka Foretova, Hervé Ghesquières, Bengt Glimelius, Thomas M Habermann, Jonathan N Hofmann, Qing Lan, Mark Liebow, Anne Lincoln, Marc Maynadie, James McKay, Mads Melbye[,,,], Lucia Miligi, Roger L Milne, Thierry J Molina, Lindsay M Morton, Kari E North, Kenneth Offit, Marina Padoan, Sara Piro, Alpa V Patel, Mark P Purdue, Vignesh Ravichandran, Elio Riboli, Richard K Severson, Melissa C Southey, Anthony Staines, Lesley F Tinker, Ruth C Travis, Sophia S Wang, Elisabete Weiderpass, Stephanie Weinstein, Tongzhang Zheng, Stephen J Chanock, Nathaniel Rothman, Brenda M Birmann, James R Cerhan, Sonja I Berndt

Table of Contents

Supplementary Table 1: ……………………………………………………………………………………page 2

Supplementary Table 2: ……………………………………………………………………………………page 3

Supplementary Table 3: ……………………………………………………………………………………page 4

Supplementary Table 4: ……………………………………………………………………………………page 5

Supplementary Table 5: ……………………………………………………………………………………page 6

Supplementary Table 6: ……………………………………………………………………………………page 7

Supplementary Table 7: ……………………………………………………………………………………page 8

Supplementary Table 8: ……………………………………………………………………………………page 9

Supplementary Table 9: ……………………………………………………………………………………page 10

Supplementary Table 10: …………………………………………………………………………………page 11

Supplementary Table 11: …………………………………………………………………………………page 12

Supplementary Figure 1: …………………………………………………………………………………page 13

Supplementary Figure 2: …………………………………………………………………………………page 14

Supplementary Figure 3: …………………………………………………………………………………page 15

Supplementary Table 1. Genome-wide association studies (GWAS) included in the meta-analysis

Study Name	Abbreviation	Study Design	Location	CLL/SLL		DLBCL		FL		MZL		Study Reference	Genotyping Platform
				Cases	Controls	Cases	Controls	Cases	Controls	Cases	Controls		
NHL InterLymph GWAS	InterLymph	Pooled analysis consisting of clinic- and population-based cases and controls from mulitple studies*	USA, Australia, and multiple European countries	2,179	6,221	2,661	6,221	2,142	6,221	825	6,221	PMID: 26956414, PMID: 25261932, PMID: 25279986, PMID: 25569183	Illumina OmniExpress/Omni2.5
Genetic Epidemiology of CLL (GEC) Consortium	GEC	Family Study	USA	387	294	0	0	0	0	0	0	PMID: 21131588	Affymetrix 6.0
Groupe d'Etude des Lymphomes de l'Adulte (GELA)/European Prospective Investigation into Cancer, Chronic Diseases, Nutrition and Lifestyles (EPIC)	GELA/EPIC	Cases from randomized clinical trials with population-based controls from EPIC	France, multiple European countries	0	0	549	525	0	0	0	0	PMID: 22118442, PMID: 9126529	Illumina HumanHap 610K and 660W
Mayo Clinic Case-Control Study of NHL and CLL and Iowa-Mayo SPORE Molecular Epidemiology Resource	MAYO-DLBCL	Clinic-based case-control study and clinic-based registry	USA	0	0	393	172	0	0	0	0	PMID: 21686124	Illumina HumanHap 660W
Scandinavian Lymphoma Etiology Study	SCALE	Population-based case-control	Denmark and Sweden	0	0	0	0	376	791	0	0	PMID: 15687363	Illumina HumanHap 317K
Molecular Epidemiology of non-Hodgkin Lymphoma	UCSF1/NHS	Case series with population controls from the Nurses' Health Study	USA	0	0	0	0	119	349	0	0	PMID: 18636124, 24096698, 12915497	Illumina OmniExpress
Molecular Epidemiology of non-Hodgkin Lymphoma	UCSF2	Population-based case-control	USA	213	746	254	748	210	746	0	0	PMID: 19620980, 22697504	Illumina HumanCNV370-Duo
Utah Chronic Lymphocytic Leukemia Study	Utah	Clinic- and population-based cases and controls	USA	321	405	0	0	0	0	0	0		Illumina HumanHap 610K
**Total subjects included in analyses**				**3,100**	**7,666**	**3,857**	**7,666**	**2,847**	**8,107**	**825**	**6,221**		

*For additional details on study design and samples, please refer to the genome-wide association studies of CLL/SLL (PMID: 23770605), DLBCL (PMID: 25261932), FL (PMID: 25279986), and MZL (PMID: 25569183).

Supplementary Table 2. Risk of four non-Hodgkin lymphoma subtypes associated with genetically determined BMI												
	Quartile 2 vs Quartile 1			Quartile 3 vs Quartile 1			Quartile 4 vs Quartile 1			Linear		
Subtype	no. cases/ no. cntls	OR (95% CI)	p-value	no. cases/ no. cntls	OR (95% CI)	p-value	no. cases/ no. cntls	OR (95% CI)	p-value	no. cases/ no. cntls	OR (95% CI)	p-value
**DLBCL**												
Combined	973/1918	1.12 (0.99-1.26)	0.07	953/1911	1.08 (0.96-1.21)	0.23	1056/1916	1.18 (1.04-1.32)	0.007	3857/7666	1.18 (1.05-1.33)	0.005
Male	507/1313	1.12 (0.95-1.31)	0.18	475/1327	1.04 (0.88-1.23)	0.63	533/1286	1.19 (1.02-1.40)	0.03	1968/5260	1.23 (1.05-1.44)	0.01
Female	466/605	1.09 (0.91-1.30)	0.36	478/584	1.12 (0.94-1.34)	0.21	523/630	1.12 (0.94-1.34)	0.20	1889/2406	1.10 (0.92-1.31)	0.29
**FL**												
Combined	715/2027	1.03 (0.90-1.17)	0.67	728/2025	1.04 (0.91-1.18)	0.58	716/2026	1.01 (0.89-1.15)	0.85	2847/8107	1.00 (0.88-1.14)	0.95
Male	323/1299	1.03 (0.86-1.24)	0.74	322/1309	1.02 (0.85-1.22)	0.87	315/1279	1.06 (0.88-1.27)	0.57	1276/5208	1.04 (0.86-1.25)	0.69
Female	368/641	1.01 (0.83-1.22)	0.94	369/629	1.04 (0.86-1.25)	0.72	366/660	0.97 (0.80-1.17)	0.72	1452/2550	0.98 (0.81-1.18)	0.82
**CLL**												
Combined	760/1916	0.99 (0.87-1.12)	0.83	757/1916	1.01 (0.90-1.15)	0.84	817/1915	1.08 (0.95-1.22)	0.25	3100/7667	1.08 (0.95-1.21)	0.25
Male	417/1347	0.94 (0.80-1.11)	0.46	441/1354	1.04 (0.89-1.22)	0.64	491/1318	1.15 (0.98-1.34)	0.09	1794/5371	1.17 (1.00-1.37)	0.05
Female	343/569	1.05 (0.86-1.28)	0.64	316/562	1.01 (0.82-1.23)	0.96	326/597	0.99 (0.81-1.21)	0.92	1306/2296	0.97 (0.79-1.18)	0.73
**MZL**												
Combined	201/1555	0.99 (0.80-1.23)	0.93	198/1555	0.97 (0.78-1.21)	0.81	228/1555	1.09 (0.88-1.34)	0.44	825/6221	1.10 (0.89-1.36)	0.38
Male	85/1132	1.00 (0.72-1.37)	0.98	81/1134	0.99 (0.72-1.37)	0.95	85/1105	1.09 (0.79-1.50)	0.60	491/1694	1.08 (0.78-1.48)	0.66
Female	116/423	0.96 (0.72-1.29)	0.78	117/421	0.97 (0.72-1.30)	0.82	143/450	1.11 (0.84-1.47)	0.47	334/4527	1.15 (0.86-1.52)	0.35

Supplementary Table 3. Risk of four non-Hodgkin lymphoma subtypes associated with genetically determined BMI using sex-specific polygenic scores

	Sex-combined PGS			Female PGS*		Male PGS*	
**Subtype**	**no. cases/ no. cntls**	**OR (95% CI)**	**p-value**	**OR (95% CI)**	**p-value**	**OR (95% CI)**	**p-value**
**DLBCL**							
Combined	3857/7666	1.18 (1.05-1.33)	0.005	1.35 (1.12-1.62)	0.002	1.31 (1.09-1.58)	0.004
Male	1968/5260	1.23 (1.05-1.44)	0.01	1.30 (1.01-1.67)	0.05	1.39 (1.07-1.79)	0.01
Female	1889/2406	1.10 (0.92-1.31)	0.29	1.40 (1.06-1.85)	0.02	1.21 (0.92-1.60)	0.18
**FL**							
Combined	2847/8107	1.00 (0.88-1.14)	0.95	1.07 (0.88-1.32)	0.49	1.19 (0.97-1.46)	0.11
Male	1276/5208	1.04 (0.86-1.25)	0.69	0.97 (0.73-1.30)	0.84	1.12 (0.83-1.50)	0.45
Female	1452/2550	0.98 (0.81-1.18)	0.82	1.18 (0.87-1.59)	0.29	1.21 (0.89-1.64)	0.23
**CLL**							
Combined	3100/7667	1.08 (0.95-1.21)	0.25	1.07 (0.88-1.30)	0.5	1.23 (1.01-1.50)	0.04
Male	1794/5371	1.17 (1.00-1.37)	0.05	1.08 (0.84-1.39)	0.54	1.33 (1.03-1.71)	0.03
Female	1306/2296	0.97 (0.79-1.18)	0.73	1.10 (0.80-1.51)	0.57	1.11 (0.81-1.53)	0.5
**MZL**							
Combined	825/6221	1.10 (0.89-1.36)	0.38	1.22 (0.87-1.72)	0.24	0.94 (0.67-1.33)	0.74
Male	491/1694	1.08 (0.78-1.48)	0.66	1.05 (0.63-1.73)	0.86	0.95 (0.57-1.58)	0.84
Female	334/4527	1.15 (0.86-1.52)	0.35	1.41 (0.89-2.23)	0.14	0.99 (0.62-1.58)	0.97

*The correlation between the female PGS and male PGS for genetically predicted BMI was 0.56 among controls.

Supplementary Table 4. Individual study results for the risk of non-Hodgkin lymphoma subtypes associated with genetically determined BMI*

	Quartile 2 vs. Quartile 1				Quartile 3 vs. Quartile 1				Quartile 4 vs. Quartile 1				Linear			
Subtype	OR (95% CI)	p-value	p_{het}	I^2	OR (95% CI)	p-value	p_{het}	I^2	OR (95% CI)	p-value	p_{het}	I^2	OR (95% CI)	p-value	p_{het}	I^2
**Sex combined**																
**DLBCL**																
Meta-analysis	1.12 (0.99-1.26)	0.07	0.94	0.0%	1.08 (0.96-1.21)	0.23	0.33	12.7%	1.18 (1.04-1.32)	0.007	0.54	0.0%	1.18 (1.05-1.33)	0.005	0.49	0.0%
GELA	1.09 (0.77-1.55)				1.09 (0.77-1.55)				1.31 (0.93-1.84)				1.38 (0.97-1.97)			
Mayo	1.33 (0.76-2.31)				1.72 (0.99-2.98)				1.58 (0.91-2.73)				1.38 (0.81-2.35)			
UCSF2	1.15 (0.77-1.70)				0.91 (0.60-1.38)				1.00 (0.66-1.50)				0.94 (0.64-1.39)			
InterLymph	1.11 (0.97-1.27)				1.06 (0.93-1.22)				1.16 (1.01-1.32)				1.17 (1.03-1.34)			
**FL**																
Meta-analysis	1.03 (0.90-1.17)	0.67	0.86	0.0%	1.04 (0.91-1.18)	0.58	0.36	7.6%	1.01 (0.89-1.15)	0.85	0.24	28.0%	1.00 (0.88-1.14)	0.95	0.13	47.0%
SCALE	1.14 (0.78-1.66)				1.28 (0.88-1.85)				1.23 (0.84-1.79)				1.43 (0.97-2.13)			
UCSF1	1.13 (0.55-2.31)				1.59 (0.82-3.10)				1.61 (0.82-3.16)				1.24 (0.67-2.32)			
UCSF2	0.89 (0.57-1.37)				1.00 (0.66-1.52)				0.79 (0.51-1.23)				0.74 (0.48-1.14)			
InterLymph	1.03 (0.89-1.19)				0.99 (0.85-1.15)				0.99 (0.85-1.15)				0.98 (0.88-1.14)			
**CLL**																
Meta-analysis	0.99 (0.87-1.12)	0.83	0.99	0.0%	1.01 (0.90-1.15)	0.84	0.36	7.2%	1.08 (0.95-1.22)	0.25	0.56	0.0%	1.08 (0.95-1.21)	0.25	0.68	0.0%
Mayo	0.92 (0.60-1.42)				0.89 (0.58-1.37)				0.95 (0.62-1.47)				1.01 (0.66-1.54)			
Utah	1.00 (0.64-1.56)				1.41 (0.92-2.15)				1.04 (0.67-1.63)				1.04 (0.67-1.60)			
UCSF2	0.97 (0.63-1.48)				0.86 (0.56-1.32)				1.41 (0.94-2.11)				1.38 (0.91-2.09)			
InterLymph	1.00 (0.86-1.15)				1.01 (0.88-1.16)				1.06 (0.92-1.22)				1.06 (0.92-1.22)			
**Male**																
**DLBCL**																
Meta-analysis	1.12 (0.95-1.31)	0.18	0.13	47.8%	1.04 (0.88-1.23)	0.63	0.15	43.4%	1.19 (1.02-1.40)	0.03	0.45	0.0%	1.23 (1.05-1.44)	0.01	0.76	0.0%
GELA	1.06 (0.63-1.77)				0.98 (0.59-1.64)				1.00 (0.61-1.66)				1.14 (0.68-1.93)			
Mayo	2.44 (1.08-5.54)				2.06 (0.93-4.58)				1.75 (0.79-3.85)				1.21 (0.55-2.66)			
UCSF2	0.75 (0.44-1.28)				0.68 (0.40-1.17)				0.90 (0.53-1.52)				0.95 (0.57-1.59)			
InterLymph	1.13 (0.94-1.36)				1.06 (0.88-1.28)				1.24 (1.03-1.49)				1.28 (1.07-1.53)			
**FL**																
Meta-analysis	1.03 (0.86-1.24)	0.74	0.83	0.0%	1.02 (0.85-1.22)	0.87	0.51	0.0%	1.06 (0.88-1.27)	0.57	0.64	0.0%	1.04 (0.86-1.25)	0.69	0.36	1.3%
SCALE	1.17 (0.67-2.06)				1.28 (0.74-2.21)				1.04 (0.60-1.79)				1.14 (0.65-1.98)			
UCSF2	0.91 (0.51-1.62)				0.79 (0.44-1.43)				0.80 (0.43-1.48)				0.69 (0.38-1.25)			
InterLymph	1.03 (0.84-1.27)				1.01 (0.82-1.25)				1.09 (0.89-1.34)				1.08 (0.88-1.32)			
**CLL**																
Meta-analysis	0.94 (0.80-1.11)	0.46	0.84	0.0%	1.04 (0.89-1.22)	0.64	0.39	14.0%	1.15 (0.98-1.34)	0.09	0.81	0.0%	1.17 (1.00-1.37)	0.05	0.95	0.0%
Mayo	0.75 (0.43-1.29)				0.82 (0.48-1.42)				0.97 (0.57-1.65)				1.05 (0.62-1.77)			
Utah	0.89 (0.51-1.57)				1.14 (0.66-1.97)				1.07 (0.61-1.89)				1.07 (0.61-1.87)			
UCSF2	1.00 (0.56-1.76)				0.69 (0.38-1.24)				1.40 (0.82-2.39)				1.26 (0.74-2.15)			
InterLymph	0.97 (0.80-1.16)				1.10 (0.91-1.31)				1.15 (0.96-1.38)				1.18 (0.99-1.37)			
**Female**																
**DLBCL**																
Meta-analysis	1.09 (0.91-1.30)	0.36	0.18	39.1%	1.12 (0.94-1.34)	0.21	0.74	0.0%	1.12 (0.94-1.34)	0.20	0.48	0.0%	1.10 (0.92-1.31)	0.29	0.48	0.0%
GELA	1.12 (0.67-1.88)				1.32 (0.77-2.26)				1.58 (0.95-2.63)				1.48 (0.87-2.51)			
Mayo	0.71 (0.33-1.56)				1.38 (0.64-2.96)				1.36 (0.64-2.90)				1.51 (0.73-3.11)			
UCSF2	1.94 (1.07-3.52)				1.34 (0.71-2.55)				1.13 (0.60-2.14)				0.93 (0.51-1.68)			
InterLymph	1.04 (0.84-1.28)				1.06 (0.86-1.30)				1.05 (0.85-1.28)				1.05 (0.85-1.28)			
**FL**																
Meta-analysis	1.01 (0.83-1.22)	0.94	0.76	0.0%	1.04 (0.86-1.25)	0.72	0.48	0.0%	0.97 (0.80-1.17)	0.72	0.23	31.2%	0.98 (0.81-1.18)	0.82	0.06	63.7%
SCALE	1.14 (0.68-1.90)				1.27 (0.76-2.14)				1.45 (0.86-2.43)				1.83 (1.04-3.22)			
UCSF2	0.83 (0.43-1.60)				1.29 (0.71-2.35)				0.78 (0.41-1.49)				0.80 (0.43-1.51)			
InterLymph	1.01 (0.81-1.25)				0.97 (0.78-1.21)				0.92 (0.75-1.15)				0.92 (0.74-1.13)			
**CLL**																
Meta-analysis	1.05 (0.86-1.28)	0.64	0.89	0.0%	1.01 (0.82-1.23)	0.96	0.14	44.6%	0.99 (0.81-1.21)	0.92	0.61	0.0%	0.97 (0.79-1.18)	0.73	0.41	0.0%
Mayo	1.31 (0.64-2.67)				1.02 (0.50-2.08)				0.90 (0.43-1.92)				0.95 (0.46-1.95)			
Utah	1.21 (0.58-2.53)				2.03 (1.02-4.03)				1.03 (0.49-2.17)				1.02 (0.51-2.03)			
UCSF2	0.96 (0.50-1.84)				1.26 (0.66-2.40)				1.48 (0.79-2.80)				1.69 (0.85-3.35)			
InterLymph	1.02 (0.81-1.28)				0.90 (0.71-1.13)				0.94 (0.75-1.18)				0.90 (0.72-1.13)			

*Genetically determined BMI based on sex-combined PGS

Supplementary Table 5. Risk of four non-Hodgkin lymphoma subtypes associated with genetically determined WHR adjusted for BMI (WHRadjBMI)*

	Quartile 2 vs Quartile 1			Quartile 3 vs Quartile 1			Quartile 4 vs Quartile 1			Linear		
**Subtype**	**no. cases/ no. cntls**	**OR (95% CI)**	**p-value**	**no. cases/ no. cntls**	**OR (95% CI)**	**p-value**	**no. cases/ no. cntls**	**OR (95% CI)**	**p-value**	**no. cases/ no. cntls**	**OR (95% CI)**	**p-value**
**DLBCL**												
Combined	924/1905	1.01 (0.89-1.13)	0.92	972/1933	1.03 (0.92-1.16)	0.60	953/1909	1.04 (0.93-1.18)	0.48	3857/7666	1.05 (0.89-1.24)	0.54
Male	467/1319	0.96 (0.82-1.13)	0.66	495/1314	1.02 (0.87-1.20)	0.80	489/1345	0.98 (0.83-1.15)	0.76	1968/5260	0.98 (0.79-1.23)	0.87
Female	457/586	1.05 (0.88-1.26)	0.57	477/619	1.04 (0.87-1.24)	0.67	464/564	1.12 (0.94-1.34)	0.20	1889/2406	1.14 (0.88-1.46)	0.32
**FL**												
Combined	754/2026	1.18 (1.03-1.34)	0.01	736/2026	1.16 (1.02-1.32)	0.03	703/2027	1.14 (0.99-1.30)	0.06	2847/8107	1.19 (1.00-1.43)	0.06
Male	327/1317	1.06 (0.88-1.27)	0.55	330/1285	1.09 (0.91-1.32)	0.34	312/1330	1.05 (0.87-1.26)	0.63	1276/5208	1.19 (0.92-1.54)	0.19
Female	393/622	1.27 (1.05-1.53)	0.01	382/654	1.22 (1.01-1.47)	0.04	349/610	1.15 (0.95-1.40)	0.15	1452/2550	1.10 (0.84-1.44)	0.47
**CLL**												
Combined	771/1916	1.02 (0.90-1.15)	0.81	796/1916	1.06 (0.94-1.20)	0.34	752/1917	1.00 (0.89-1.13)	0.99	3100/7667	1.05 (0.88-1.24)	0.59
Male	438/1362	0.96 (0.82-1.13)	0.65	485/1328	1.09 (0.94-1.28)	0.27	418/1372	0.91 (0.78-1.07)	0.26	1794/5371	1.00 (0.80-1.25)	0.99
Female	333/554	1.11 (0.91-1.35)	0.30	311/588	1.02 (0.83-1.24)	0.88	334/545	1.16 (0.96-1.42)	0.13	1306/2296	1.13 (0.86-1.49)	0.37
**MZL**												
Combined	217/1555	1.05 (0.86-1.30)	0.63	203/1555	1.00 (0.81-1.24)	0.97	185/1555	0.96 (0.77-1.19)	0.71	825/6221	0.91 (0.68-1.23)	0.56
Male	80/1145	0.77 (0.57-1.05)	0.10	79/1128	0.77 (0.57-1.05)	0.10	67/1155	0.67 (0.49-0.93)	0.02	334/4527	0.60 (0.38-0.94)	0.03
Female	137/410	1.40 (1.06-1.87)	0.02	124/427	1.26 (0.94-1.68)	0.13	118/400	1.29 (0.96-1.74)	0.09	491/1694	1.25 (0.84-1.88)	0.28

*Genetically determined WHRadjBMI based on sex-combined PGS

Supplementary Table 6. Risk of four non-Hodgkin lymphoma subtypes associated with genetically determined WHR (unadjusted)*

Subtype	Quartile 2 vs Quartile 1 no. cases/ no. cntls	OR (95% CI)	p-value	Quartile 3 vs Quartile 1 no. cases/ no. cntls	OR (95% CI)	p-value	Quartile 4 vs Quartile 1 no. cases/ no. cntls	OR (95% CI)	p-value	Linear no. cases/ no. cntls	OR (95% CI)	p-value
**DLBCL**												
Combined	975/1912	1.09 (0.97-1.22)	0.17	974/1917	1.08 (0.96-1.21)	0.22	967/1919	1.11 (0.98-1.25)	0.10	3857/7666	1.15 (0.94-1.41)	0.18
Male	498/1322	1.09 (0.93-1.28)	0.29	497/1282	1.10 (0.93-1.29)	0.27	494/1349	1.08 (0.92-1.27)	0.34	1968/5260	1.14 (0.87-1.50)	0.34
Female	477/590	1.07 (0.89-1.27)	0.48	477/635	1.03 (0.86-1.22)	0.79	473/570	1.14 (0.96-1.37)	0.15	1889/2406	1.15 (0.85-1.57)	0.37
**FL**												
Combined	704/2026	1.04 (0.91-1.18)	0.56	695/2025	1.02 (0.90-1.17)	0.73	754/2027	1.12 (0.99-1.28)	0.08	2847/8107	1.16 (0.93-1.45)	0.18
Male	320/1309	1.02 (0.84-1.22)	0.88	306/1274	1.03 (0.85-1.24)	0.79	327/1334	1.03 (0.86-1.24)	0.77	1276/5208	1.06 (0.77-1.45)	0.74
Female	362/630	1.09 (0.90-1.31)	0.40	357/664	1.01 (0.84-1.22)	0.9	387/606	1.20 (0.99-1.45)	0.06	1452/2550	1.18 (0.85-1.64)	0.31
**CLL**												
Combined	780/1916	1.02 (0.91-1.16)	0.71	737/1915	0.95 (0.84-1.08)	0.44	782/1916	1.03 (0.91-1.16)	0.65	3100/7667	1.02 (0.82-1.25)	0.88
Male	465/1361	1.07 (0.91-1.25)	0.41	438/1309	1.04 (0.89-1.22)	0.60	447/1373	1.02 (0.87-1.19)	0.84	1794/5371	1.05 (0.80-1.37)	0.75
Female	315/555	0.95 (0.78-1.15)	0.58	299/606	0.83 (0.69-1.02)	0.07	335/543	1.06 (0.88-1.29)	0.54	1306/2296	1.01 (0.72-1.41)	0.97
**MZL**												
Combined	207/1555	0.97 (0.79-1.20)	0.78	211/1555	1.00 (0.81-1.23)	0.97	181/1555	0.90 (0.72-1.11)	0.32	825/6221	0.85 (0.59-1.24)	0.40
Male	78/1141	0.81 (0.59-1.10)	0.18	90/1106	0.99 (0.73-1.35)	0.97	65/1161	0.67 (0.49-0.94)	0.02	491/1694	0.58 (0.34-1.02)	0.06
Female	129/414	1.11 (0.84-1.47)	0.48	121/449	0.99 (0.74-1.32)	0.95	116/394	1.10 (0.82-1.47)	0.54	334/4527	1.13 (0.68-1.86)	0.64

*Genetically determined WHR based on sex-combined PGS

Supplementary Table 7. Risk of four non-Hodgkin lymphoma subtypes associated with genetically determined WHR adjusted for BMI (WHRadjBMI) using sex-specific polygenic scores

		Sex-combined PGS		Female PGS		Male PGS	
**Subtype**	**no. cases/ no. cntls**	**OR (95% CI)**	**p-value**	**OR (95% CI)**	**p-value**	**OR (95% CI)**	**p-value**
**DLBCL**							
Combined	3857/7666	1.05 (0.89-1.24)	0.54	1.02 (0.89-1.16)	0.82	0.93 (0.70-1.25)	0.63
Male	1968/5260	0.98 (0.79-1.23)	0.87	1.01 (0.84-1.21)	0.94	0.96 (0.64-1.43)	0.84
Female	1889/2406	1.14 (0.88-1.46)	0.32	1.01 (0.82-1.23)	0.95	0.94 (0.61-1.47)	0.8
**FL**							
Combined	2847/8107	1.19 (1.00-1.43)	0.06	1.12 (0.97-1.29)	0.14	1.43 (1.03-1.98)	0.03
Male	1276/5208	1.19 (0.92-1.54)	0.19	1.08 (0.88-1.33)	0.47	0.98 (0.62-1.54)	0.92
Female	1452/2550	1.10 (0.84-1.44)	0.47	1.10 (0.89-1.37)	0.38	2.14 (1.32-3.47)	0.002
**CLL**							
Combined	3100/7667	1.05 (0.88-1.24)	0.59	0.97 (0.84-1.11)	0.66	1.05 (0.78-1.43)	0.75
Male	1794/5371	1.00 (0.80-1.25)	0.99	0.91 (0.76-1.09)	0.3	0.99 (0.67-1.47)	0.97
Female	1306/2296	1.13 (0.86-1.49)	0.37	1.06 (0.85-1.32)	0.63	1.18 (0.72-1.94)	0.51
**MZL**							
Combined	825/6221	0.91 (0.68-1.23)	0.56	1.05 (0.83-1.34)	0.68	0.66 (0.38-1.12)	0.12
Male	334/4527	0.60 (0.38-0.94)	0.03	0.78 (0.55-1.13)	0.19	0.47 (0.21-1.05)	0.07
Female	491/1694	1.25 (0.84-1.88)	0.28	1.32 (0.95-1.83)	0.1	0.84 (0.41-1.74)	0.64

*The correlation between the female PGS and male PGS for genetically predicted WHRadjBMI was 0.27 among controls.

Supplementary Table 8. Risk of four non-Hodgkin lymphoma subtypes associated with genetically determined WHR (unadjusted) using sex-specific polygenic scores

		Sex-combined PGS		Female PGS		Male PGS	
Subtype	no. cases/ no. cntls	OR (95% CI)	p-value	OR (95% CI)	p-value	OR (95% CI)	p-value
**DLBCL**							
Combined	3857/7666	1.15 (0.94-1.41)	0.18	1.20 (1.00-1.42)	0.05	1.17 (0.83-1.65)	0.37
Male	1968/5260	1.14 (0.87-1.50)	0.34	1.22 (0.96-1.54)	0.11	1.43 (0.89-2.28)	0.14
Female	1889/2406	1.15 (0.85-1.57)	0.37	1.14 (0.87-1.48)	0.35	0.96 (0.58-1.61)	0.89
**FL**							
Combined	2847/8107	1.16 (0.93-1.45)	0.18	1.22 (1.01-1.48)	0.04	1.37 (0.95-2.00)	0.1
Male	1276/5208	1.06 (0.77-1.45)	0.74	1.21 (0.92-1.60)	0.18	1.31 (0.77-2.23)	0.32
Female	1452/2550	1.18 (0.85-1.64)	0.31	1.12 (0.84-1.48)	0.45	1.28 (0.74-2.22)	0.38
**CLL**							
Combined	3100/7667	1.02 (0.82-1.25)	0.88	1.14 (0.95-1.36)	0.17	0.98 (0.69-1.40)	0.91
Male	1794/5371	1.05 (0.80-1.37)	0.75	1.16 (0.91-1.47)	0.23	1.00 (0.64-1.59)	0.99
Female	1306/2296	1.01 (0.72-1.41)	0.97	1.12 (0.84-1.49)	0.44	1.00 (0.57-1.75)	0.99
**MZL**							
Combined	825/6221	0.85 (0.59-1.24)	0.40	0.98 (0.72-1.35)	0.92	0.67 (0.36-1.25)	0.21
Male	491/1694	0.58 (0.34-1.02)	0.06	0.72 (0.44-1.17)	0.18	0.54 (0.21-1.37)	0.19
Female	334/4527	1.13 (0.68-1.86)	0.64	1.23 (0.81-1.88)	0.34	0.84 (0.36-1.92)	0.67

*The correlation between the female PGS and male PGS for genetically predicted WHR was 0.21 among controls.

Supplementary Table 9. Individual study results for the risk of three non-Hodgkin lymphoma subtypes associated with genetically determined WHRadjBMI*

	Quartile 2 vs. Quartile 1				Quartile 3 vs. Quartile 1				Quartile 4 vs. Quartile 1				Linear			
**Subtype**	**OR (95% CI)**	**p-value**	p_{het}	I^2	**OR (95% CI)**	**p-value**	p_{het}	I^2	**OR (95% CI)**	**p-value**	p_{het}	I^2	**OR (95% CI)**	**p-value**	p_{het}	I^2
**Sex combined**																
**DLBCL**																
Meta-analysis	1.01 (0.89-1.13)	0.92	0.002	79.7%	1.03 (0.92-1.16)	0.60	0.09	53.4%	1.04 (0.93-1.18)	0.48	0.14	46.0%	1.05 (0.89-1.24)	0.54	0.56	0.0%
GELA	1.12 (0.79-1.58)				0.88 (0.63-1.24)				0.98 (0.69-1.40)				1.15 (0.70-1.90)			
Mayo	0.35 (0.20-0.61)				0.58 (0.34-0.99)				0.57 (0.34-0.97)				0.64 (0.31-1.33)			
UCSF2	1.17 (0.78-1.77)				1.24 (0.83-1.87)				1.12 (0.74-1.69)				1.16 (0.65-2.07)			
InterLymph	1.04 (0.91-1.19)				1.08 (0.94-1.23)				1.09 (0.95-1.25)				1.06 (0.88-1.29)			
**FL**																
Meta-analysis	1.18 (1.03-1.34)	0.01	0.04	64.5%	1.16 (1.02-1.32)	0.03	0.25	27.7%	1.14 (0.99-1.30)	0.06	0.09	54.0%	1.19 (1.00-1.43)	0.06	0.14	44.7%
SCALE	0.92 (0.64-1.34)				0.96 (0.66-1.39)				0.99 (0.68-1.43)				0.94 (0.55-1.58)			
UCSF1	2.08 (1.04-4.18)				1.13 (0.54-2.36)				2.26 (1.14-4.50)				2.47 (0.99-6.16)			
UCSF2	0.77 (0.50-1.19)				0.83 (0.54-1.28)				0.83 (0.54-1.28)				0.77 (0.42-1.43)			
InterLymph	1.25 (1.08-1.45)				1.24 (1.07-1.45)				1.17 (1.00-1.36)				1.26 (1.02-1.55)			
**CLL**																
Meta-analysis	1.02 (0.90-1.15)	0.81	0.36	6.8%	1.06 (0.94-1.20)	0.34	0.65	0.0%	1.00 (0.89-1.13)	0.99	0.83	0.0%	1.05 (0.88-1.24)	0.59	0.31	16.3%
Mayo	1.07 (0.70-1.64)				0.96 (0.62-1.48)				0.87 (0.56-1.35)				0.79 (0.42-1.49)			
Utah	0.96 (0.61-1.51)				1.20 (0.78-1.85)				1.15 (0.75-1.77)				1.67 (0.90-3.08)			
UCSF2	0.70 (0.46-1.08)				0.86 (0.57-1.30)				1.07 (0.71-1.60)				1.28 (0.71-2.29)			
InterLymph	1.06 (0.92-1.22)				1.08 (0.94-1.25)				0.99 (0.86-1.15)				1.01 (0.83-1.22)			
**Male**																
**DLBCL**																
Meta-analysis	0.96 (0.82-1.13)	0.66	0.09	53.1%	1.02 (0.87-1.20)	0.80	0.02	69.8%	0.98 (0.83-1.15)	0.76	0.35	8.5%	0.98 (0.79-1.23)	0.87	0.64	0.0%
GELA	0.98 (0.58-1.65)				0.74 (0.45-1.22)				1.00 (0.60-1.66)				0.94 (0.45-1.98)			
Mayo	0.33 (0.14-0.80)				0.33 (0.14-0.78)				0.48 (0.22-1.07)				0.50 (0.17-1.45)			
UCSF2	1.22 (0.72-2.08)				1.37 (0.80-2.34)				0.91 (0.52-1.60)				0.95 (0.44-2.03)			
InterLymph	0.98 (0.82-1.18)				1.09 (0.90-1.30)				1.02 (0.85-1.22)				1.03 (0.80-1.33)			
**FL**																
Meta-analysis	1.06 (0.88-1.27)	0.55	0.53	0.0%	1.09 (0.91-1.32)	0.34	0.37	0.1%	1.05 (0.87-1.26)	0.63	0.72	0.0%	1.19 (0.92-1.54)	0.19	0.67	0.0%
SCALE	1.05 (0.60-1.83)				1.42 (0.81-2.47)				1.06 (0.61-1.87)				1.33 (0.60-2.99)			
UCSF2	0.77 (0.43-1.38)				0.79 (0.43-1.43)				0.84 (0.47-1.49)				0.84 (0.37-1.91)			
InterLymph	1.10 (0.90-1.36)				1.10 (0.89-1.36)				1.08 (0.87-1.33)				1.22 (0.92-1.63)			
**CLL**																
Meta-analysis	0.96 (0.82-1.13)	0.65	0.40	0.0%	1.09 (0.94-1.28)	0.27	0.64	0.0%	0.91 (0.78-1.07)	0.26	0.59	0.0%	1.00 (0.80-1.25)	0.99	0.19	37.4%
Mayo	0.64 (0.37-1.09)				0.89 (0.52-1.54)				0.66 (0.38-1.13)				0.57 (0.26-1.27)			
Utah	0.97 (0.55-1.71)				1.48 (0.86-2.55)				1.04 (0.60-1.81)				1.77 (0.81-3.85)			
UCSF2	0.84 (0.48-1.48)				1.09 (0.63-1.88)				1.06 (0.61-1.85)				1.42 (0.64-3.14)			
InterLymph	1.02 (0.85-1.23)				1.08 (0.90-1.30)				0.92 (0.76-1.10)				0.96 (0.75-1.24)			
**Female**																
**DLBCL**																
Meta-analysis	1.06 (0.88-1.26)	0.57	0.04	64.9%	1.04 (0.87-1.24)	0.67	0.91	0.0%	1.12 (0.94-1.34)	0.20	0.35	7.2%	1.14 (0.88-1.46)	0.32	0.70	0.0%
GELA	1.12 (0.67-1.86)				0.94 (0.56-1.56)				1.01 (0.60-1.71)				1.46 (0.69-3.09)			
Mayo	0.36 (0.17-0.75)				0.86 (0.42-1.73)				0.62 (0.29-1.31)				0.80 (0.29-2.20)			
UCSF2	1.12 (0.58-2.13)				1.10 (0.59-2.06)				1.42 (0.76-2.64)				1.54 (0.63-3.77)			
InterLymph	1.12 (0.92-1.37)				1.07 (0.87-1.31)				1.16 (0.95-1.43)				1.09 (0.82-1.45)			
**FL**																
Meta-analysis	1.27 (1.05-1.53)	0.01	0.04	68.6%	1.22 (1.01-1.47)	0.04	0.03	71.0%	1.15 (0.95-1.40)	0.15	0.22	33.5%	1.10 (0.84-1.44)	0.47	0.15	46.6%
SCALE	0.85 (0.51-1.40)				0.71 (0.42-1.18)				0.88 (0.54-1.44)				0.68 (0.33-1.36)			
UCSF2	0.76 (0.39-1.45)				0.87 (0.47-1.61)				0.81 (0.42-1.55)				0.68 (0.27-1.74)			
InterLymph	1.45 (1.17-1.80)				1.39 (1.12-1.73)				1.27 (1.02-1.58)				1.27 (0.94-1.73)			
**CLL**																
Meta-analysis	1.11 (0.91-1.35)	0.30	0.02	69.4%	1.02 (0.83-1.24)	0.88	0.36	7.6%	1.16 (0.96-1.42)	0.13	0.91	0.0%	1.13 (0.86-1.49)	0.37	0.93	0.0%
Mayo	2.58 (1.26-5.31)				1.12 (0.54-2.32)				1.48 (0.69-3.15)				1.42 (0.49-4.15)			
Utah	0.92 (0.44-1.92)				0.89 (0.43-1.81)				1.28 (0.64-2.57)				1.45 (0.53-3.95)			
UCSF2	0.55 (0.28-1.07)				0.59 (0.30-1.13)				1.09 (0.60-1.96)				1.11 (0.46-2.67)			
InterLymph	1.13 (0.90-1.41)				1.09 (0.87-1.36)				1.14 (0.91-1.43)				1.09 (0.86-1.49)			

*Genetically determined WHRadjBMI based on sex-combined PGS

Supplementary Table 10. Individual study results for the risk of three non-Hodgkin lymphoma subtypes associated with genetically determined WHR (unadjusted)*

	Quartile 2 vs. Quartile 1				Quartile 3 vs. Quartile 1				Quartile 4 vs. Quartile 1				Linear			
**Subtype**	**OR (95% CI)**	**p-value**	**p_{het}**	**I^2**	**OR (95% CI)**	**p-value**	**p_{het}**	**I^2**	**OR (95% CI)**	**p-value**	**p_{het}**	**I^2**	**OR (95% CI)**	**p-value**	**p_{het}**	**I^2**
**Sex combined**																
**DLBCL**																
Meta-analysis	1.09 (0.97-1.22)	0.17	0.18	38.5%	1.08 (0.96-1.21)	0.22	0.32	15.2%	1.11 (0.98-1.25)	0.10	0.19	36.3%	1.15 (0.94-1.41)	0.18	0.56	0.0%
GELA	1.26 (0.88-1.78)				1.30 (0.92-1.83)				1.12 (0.79-1.60)				1.43 (0.77-2.66)			
Mayo	0.63 (0.37-1.08)				0.72 (0.41-1.24)				0.68 (0.40-1.17)				0.82 (0.32-2.10)			
UCSF2	0.99 (0.65-1.52)				1.20 (0.80-1.81)				1.44 (0.96-2.14)				1.62 (0.80-3.25)			
InterLymph	1.11 (0.97-1.27)				1.06 (0.93-1.22)				1.10 (0.96-1.27)				1.09 (0.87-1.38)			
**FL**																
Meta-analysis	1.04 (0.91-1.18)	0.56	0.28	22.5%	1.02 (0.90-1.17)	0.73	0.55	0.0%	1.12 (0.99-1.28)	0.08	0.02	69.7%	1.16 (0.93-1.45)	0.18	0.01	73.7%
SCALE	0.81 (0.56-1.18)				0.93 (0.65-1.34)				0.92 (0.64-1.32)				0.91 (0.47-1.74)			
UCSF1	0.84 (0.42-1.70)				1.23 (0.64-2.40)				1.57 (0.82-3.01)				2.91 (1.01-8.40)			
UCSF2	0.85 (0.56-1.29)				0.80 (0.52-1.22)				0.62 (0.40-0.98)				0.40 (0.19-0.87)			
InterLymph	1.12 (0.96-1.30)				1.06 (0.91-1.24)				1.22 (1.05-1.41)				1.29 (1.00-1.66)			
**CLL**																
Meta-analysis	1.02 (0.91-1.16)	0.71	0.36	6.5%	0.95 (0.84-1.08)	0.44	0.51	0.0%	1.03 (0.91-1.16)	0.65	0.80	0.0%	1.02 (0.82-1.25)	0.88	0.47	0.0%
Mayo	0.96 (0.62-1.47)				0.99 (0.64-1.52)				0.98 (0.64-1.51)				0.89 (0.42-1.88)			
Utah	1.04 (0.68-1.60)				0.82 (0.52-1.30)				1.22 (0.80-1.86)				1.11 (0.53-2.32)			
UCSF2	0.72 (0.47-1.09)				0.73 (0.48-1.11)				1.12 (0.75-1.67)				1.72 (0.85-3.49)			
InterLymph	1.07 (0.93-1.23)				0.99 (0.86-1.14)				1.00 (0.87-1.16)				0.96 (0.75-1.22)			
**Male**																
**DLBCL**																
Meta-analysis	1.10 (0.93-1.28)	0.29	0.33	13.5%	1.10 (0.93-1.29)	0.27	0.94	0.0%	1.08 (0.92-1.27)	0.34	0.48	0.0%	1.14 (0.87-1.50)	0.34	0.66	0.0%
GELA	1.46 (0.86-2.48)				1.15 (0.69-1.91)				1.44 (0.86-2.42)				1.66 (0.68-4.07)			
Mayo	0.78 (0.35-1.76)				0.84 (0.36-1.95)				0.75 (0.34-1.63)				1.09 (0.27-4.34)			
UCSF2	0.76 (0.43-1.33)				1.08 (0.63-1.86)				1.24 (0.74-2.09)				1.67 (0.67-4.15)			
InterLymph	1.11 (0.93-1.34)				1.10 (0.92-1.33)				1.05 (0.87-1.26)				1.05 (0.87-1.50)			
**FL**																
Meta-analysis	1.02 (0.84-1.22)	0.88	0.59	0.0%	1.03 (0.85-1.24)	0.79	0.74	0.0%	1.03 (0.86-1.24)	0.77	0.06	63.7%	1.06 (0.77-1.45)	0.74	0.07	61.8%
SCALE	1.07 (0.61-1.86)				1.06 (0.62-1.80)				0.82 (0.47-1.43)				1.03 (0.38-2.80)			
UCSF2	0.77 (0.44-1.35)				0.83 (0.46-1.48)				0.55 (0.29-1.02)				0.33 (0.12-0.95)			
InterLymph	1.05 (0.85-1.29)				1.05 (0.85-1.30)				1.14 (0.92-1.40)				1.21 (0.85-1.72)			
**CLL**																
Meta-analysis	1.07 (0.91-1.25)	0.42	0.44	0.0%	1.04 (0.89-1.22)	0.60	0.43	0.0%	1.02 (0.87-1.19)	0.84	0.81	0.0%	1.05 (0.80-1.37)	0.75	0.64	0.0%
Mayo	0.92 (0.54-1.59)				0.89 (0.52-1.52)				0.94 (0.54-1.61)				0.90 (0.35-2.30)			
Utah	1.21 (0.71-2.07)				0.84 (0.46-1.53)				1.22 (0.71-2.10)				1.02 (0.41-2.57)			
UCSF2	0.71 (0.41-1.25)				0.75 (0.43-1.32)				1.19 (0.70-2.02)				1.87 (0.73-4.78)			
InterLymph	1.12 (0.93-1.34)				1.12 (0.94-1.35)				0.99 (0.82-1.19)				1.00 (0.80-1.37)			
**Female**																
**DLBCL**																
Meta-analysis	1.07 (0.89-1.27)	0.48	0.16	41.5%	1.03 (0.86-1.22)	0.79	0.29	20.0%	1.14 (0.96-1.37)	0.15	0.20	36.2%	1.15 (0.85-1.57)	0.37	0.69	0.0%
GELA	1.10 (0.65-1.84)				1.33 (0.79-2.25)				0.98 (0.58-1.67)				1.49 (0.59-3.79)			
Mayo	0.49 (0.23-1.03)				0.64 (0.32-1.32)				0.63 (0.29-1.34)				0.62 (0.17-2.27)			
UCSF2	1.47 (0.75-2.87)				1.44 (0.76-2.73)				1.80 (0.95-3.38)				1.54 (0.52-4.57)			
InterLymph	1.09 (0.89-1.34)				0.99 (0.81-1.21)				1.16 (0.95-1.43)				1.13 (0.79-1.60)			
**FL**																
Meta-analysis	1.09 (0.90-1.31)	0.4	0.10	56.6%	1.01 (0.84-1.22)	0.90	0.39	0.0%	1.20 (0.99-1.45)	0.06	0.16	46.2%	1.18 (0.85-1.64)	0.31	0.15	47.7%
SCALE	0.66 (0.39-1.11)				0.82 (0.49-1.35)				0.97 (0.60-1.57)				0.78 (0.33-1.85)			
UCSF2	0.94 (0.50-1.75)				0.77 (0.41-1.43)				0.73 (0.38-1.41)				0.51 (0.17-1.57)			
InterLymph	1.20 (0.97-1.50)				1.09 (0.88-1.35)				1.32 (1.06-1.64)				1.40 (0.97-2.03)			
**CLL**																
Meta-analysis	0.95 (0.78-1.15)	0.58	0.76	0.0%	0.83 (0.69-1.02)	0.07	0.69	0.0%	1.06 (0.88-1.29)	0.54	0.98	0.0%	1.01 (0.72-1.41)	0.97	0.82	0.0%
Mayo	1.05 (0.51-2.14)				1.22 (0.60-2.50)				1.07 (0.52-2.18)				0.88 (0.25-3.07)			
Utah	0.72 (0.34-1.54)				0.76 (0.37-1.54)				1.21 (0.63-2.36)				1.29 (0.38-4.36)			
UCSF2	0.75 (0.39-1.44)				0.69 (0.36-1.31)				1.06 (0.57-1.94)				1.56 (0.52-4.64)			
InterLymph	0.99 (0.79-1.23)				0.83 (0.66-1.04)				1.05 (0.84-1.32)				0.94 (0.64-1.39)			

*Genetically determined WHR based on sex-combined PGS

Supplementary Table 11. Risk of non-Hodgkin lymphoma associated with genetically determined BMI, WHR, and WHRadjBMI using Mendelian randomization analyses

	BMI					WHRadjBMI					WHR (unadjusted)				
**Subtype**	**OR (95%CI)**	**P**	**MR Egger intercept (±SE)**	**MR Egger P**	**Cochrane Q P**	**OR (95%CI)**	**P**	**MR Egger intercept (±SE)**	**MR Egger P**	**Cochrane Q P**	**OR (95%CI)**	**P**	**MR Egger intercept (±SE)**	**MR Egger P**	**Cochrane Q P**
**DLBCL**															
Inverse variance weighted	1.12 (1.01-1.23)	0.03			0.12	1.06 (0.89-1.27)	0.5			0.50	1.15 (0.93-1.42)	0.21			0.53
Weighted median	1.21 (1.01-1.46)	0.03				1.34 (0.99-1.81)	0.06				1.36 (0.95-1.94)	0.09			
Weighted mode	1.23 (1.02-1.48)	0.03				1.63 (0.92-2.87)	0.09				1.94 (0.94-4.00)	0.07			
MR Egger	1.04 (0.89-1.22)	0.59	0.002 (±0.002)	0.30	0.12	1.41 (0.94-2.13)	0.1	-0.006 (±0.004)	0.13	0.51	1.87 (1.04-3.35)	0.04	-0.009 (±0.005)	0.08	0.56
**FL**															
Inverse variance weighted	1.01 (0.91-1.13)	0.80			0.12	1.18 (0.96-1.45)	0.11			0.05	1.10 (0.87-1.39)	0.43			0.72
Weighted median	1.02 (0.85-1.22)	0.85				1.31 (0.94-1.82)	0.11				1.17 (0.81-1.71)	0.41			
Weighted mode	1.10 (0.90-1.34)	0.34				1.40 (0.79-2.47)	0.24				1.55 (0.69-3.46)	0.29			
MR Egger	1.14 (0.96-1.35)	0.15	-0.003 (±0.002)	0.10	0.13	1.47 (0.92-2.36)	0.11	-0.005 (±0.005)	0.32	0.05	1.23 (0.65-2.32)	0.53	-0.002 (±0.005)	0.72	0.71
**CLL**															
Inverse variance weighted	1.05 (0.95-1.17)	0.34			0.03	1.07 (0.88-1.30)	0.52			0.05	1.01 (0.80-1.27)	0.94			0.18
Weighted median	1.02 (0.84-1.23)	0.86				1.13 (0.83-1.54)	0.43				1.05 (0.72-1.53)	0.79			
Weighted mode	1.03 (0.83-1.28)	0.79				1.09 (0.61-1.96)	0.76				1.05 (0.53-2.10)	0.88			
MR Egger	1.09 (0.92-1.29)	0.32	-0.001 (±0.002)	0.60	0.03	1.09 (0.69-1.72)	0.71	-0.0005 (±0.004)	0.91	0.05	0.97 (0.51-1.82)	0.92	0.0008 (±0.005)	0.89	0.17
**MZL**															
Inverse variance weighted	1.06 (0.90-1.27)	0.48			0.79	0.97 (0.70-1.33)	0.84			0.73	0.89 (0.60-1.31)	0.54			0.74
Weighted median	1.14 (0.84-1.55)	0.40				1.25 (0.72-2.18)	0.42				0.83 (0.45-1.51)	0.54			
Weighted mode	1.06 (0.75-1.50)	0.74				1.02 (0.36-2.94)	0.96				0.78 (0.22-2.75)	0.71			
MR Egger	0.98 (0.74-1.30)	0.90	0.002 (±0.003)	0.47	0.78	1.50 (0.72-3.11)	0.28	-0.009 (±0.007)	0.19	0.74	0.90 (0.31-2.57)	0.84	-0.0002 (±0.009)	0.99	0.73

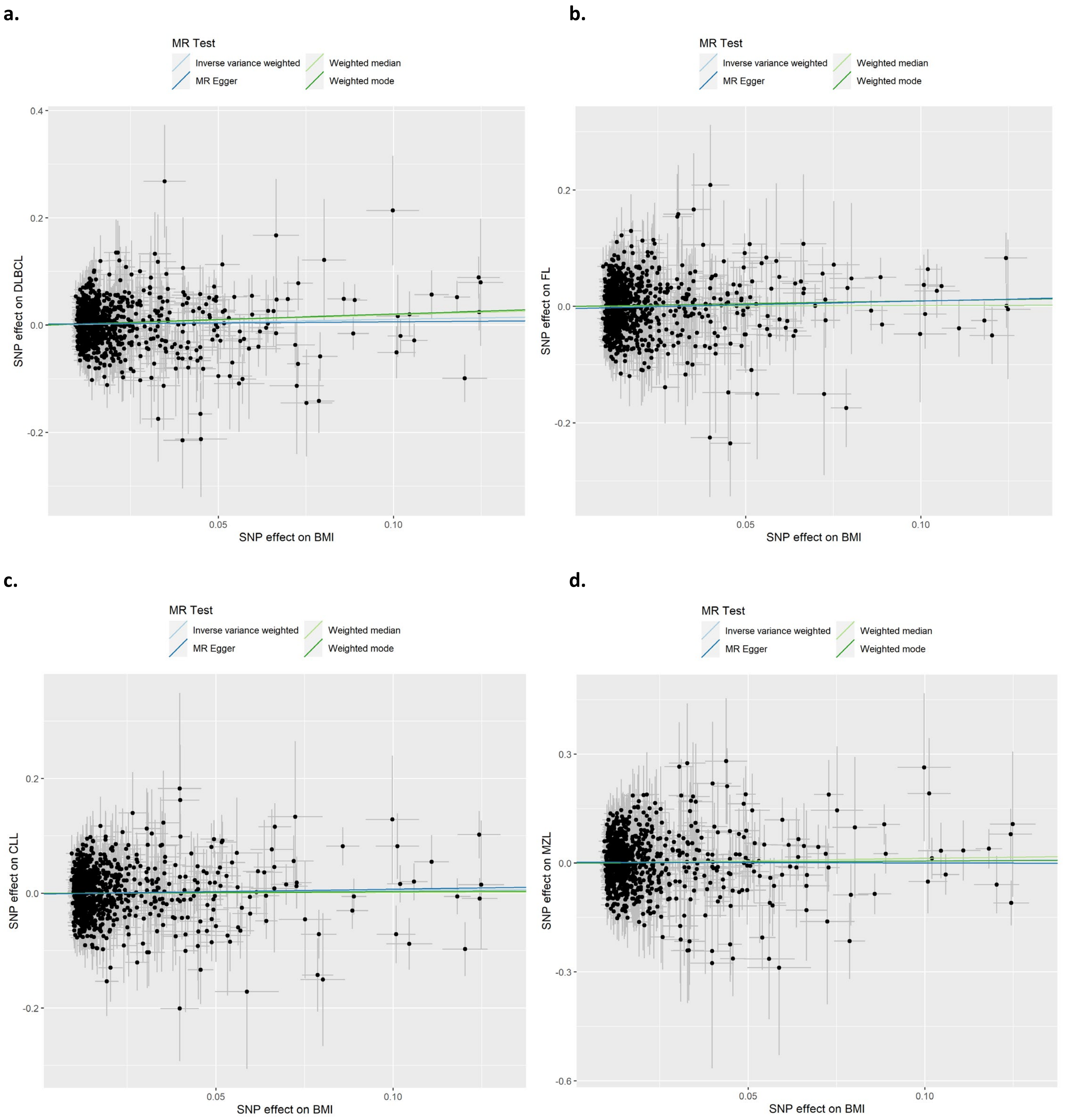

Supplementary Figure 1. Scatterplots of SNP-specific effects for the associations with BMI and (a) DLBCL, (b) FL, (c) CLL, and (d) MZL. SNP-specific vertical and horizontal bars correspond to standard errors for the NHL subtype and BMI associations, respectively. The lines represent the results of the subtype-specific Mendelian randomization regression analyses: inverse variance weighted, MR-Egger, weighted median, and weighted model.

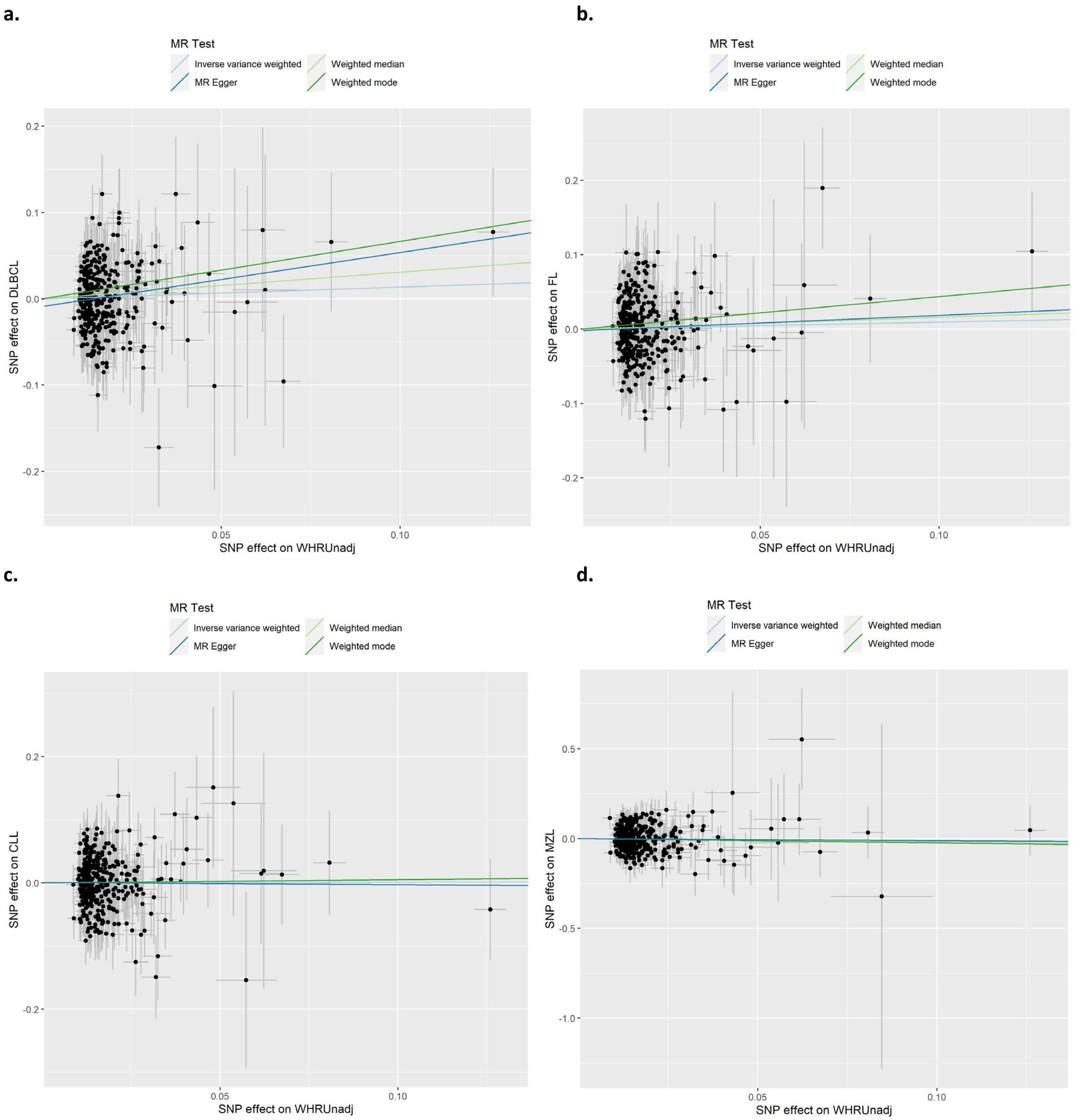

Supplementary Figure 2. Scatterplots of SNP-specific effects for the associations with WHR (unadjusted) and (a) DLBCL, (b) FL, (c) CLL, and (d) MZL. SNP-specific vertical and horizontal bars correspond to standard errors for the NHL subtype and WHR associations, respectively. The lines represent the results of the subtype-specific Mendelian randomization regression analyses: inverse variance weighted, MR-Egger, weighted median, and weighted model.

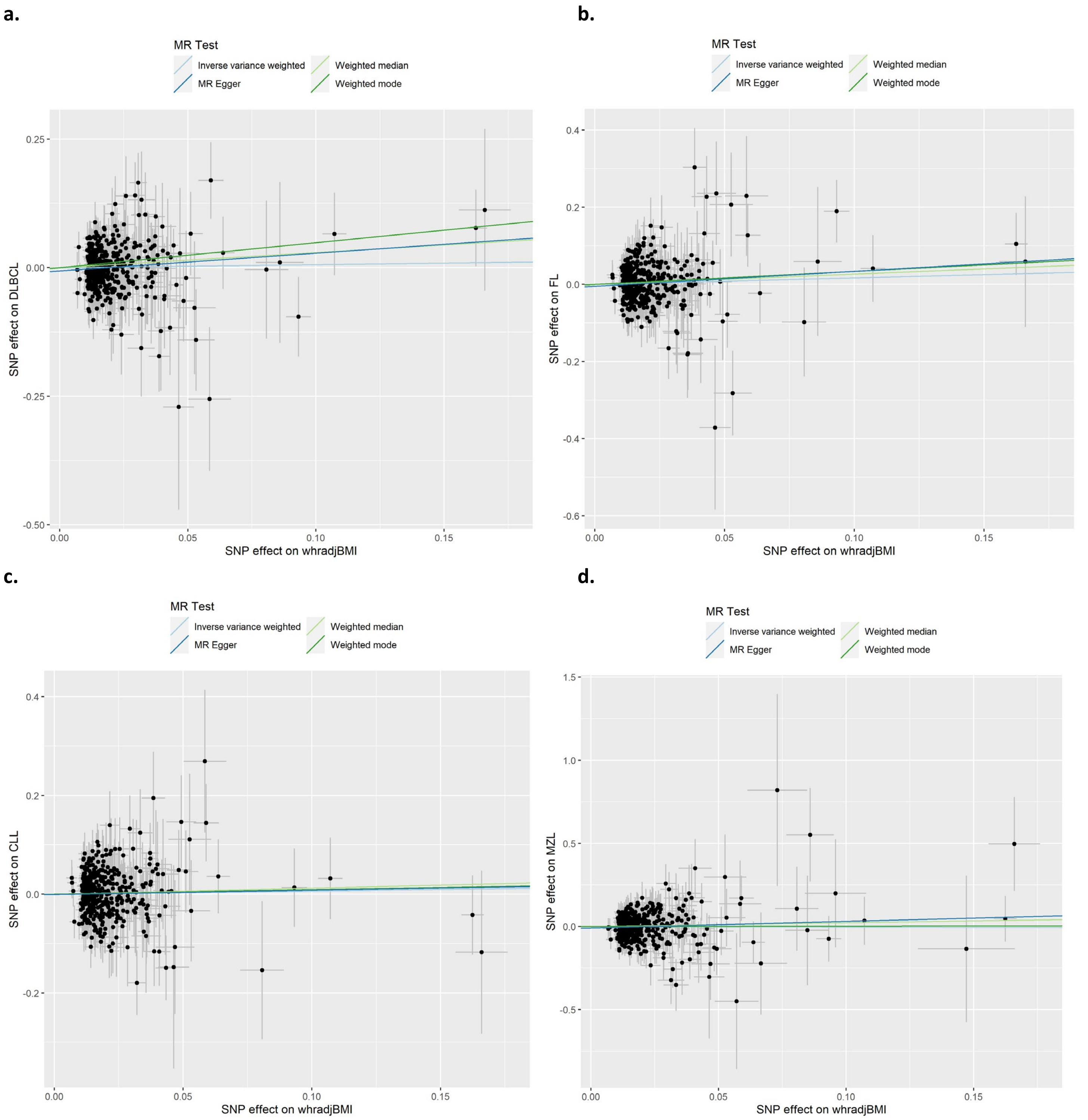

Supplementary Figure 3. Scatterplots of SNP-specific effects for the associations with WHRadjBMI and (a) DLBCL, (b) FL, (c) CLL, and (d) MZL. SNP-specific vertical and horizontal bars correspond to standard errors for the NHL subtype and WHRadjBMI associations, respectively. The lines represent the results of the subtype-specific Mendelian randomization regression analyses: inverse variance weighted, MR-Egger, weighted median, and weighted model.
